# Molecular imprinting-based indirect fluorescence detection strategy implemented on paper chip for non-fluorescent microcystin

**DOI:** 10.1038/s41467-023-42244-z

**Published:** 2023-10-17

**Authors:** Bowei Li, Ji Qi, Feng Liu, Rongfang Zhao, Maryam Arabi, Abbas Ostovan, Jinming Song, Xiaoyan Wang, Zhiyang Zhang, Lingxin Chen

**Affiliations:** 1 CAS Key Laboratory of Coastal Environmental Processes and Ecological Remediation, Yantai Institute of Coastal Zone Research, Chinese Academy of Sciences, 264003 Yantai, China; 2 Center for Ocean Mega-Science, Chinese Academy of Sciences, 266071 Qingdao, China; 3 CAS Key Laboratory of Marine Ecology and Environmental Sciences, Institute of Oceanology, Chinese Academy of Sciences, 266071 Qingdao, China; 4 Laboratory for Marine Ecology and Environmental Sciences, Qingdao National Laboratory for Marine Science and Technology, 266237 Qingdao, China; 5 School of Pharmacy, Binzhou Medical University, 264003 Yantai, China; 6 Laboratory for Marine Biology and Biotechnology, Pilot National Laboratory for Marine Science and Technology, 266237 Qingdao, China

**Keywords:** Sensors, Lab-on-a-chip, Environmental monitoring

## Abstract

Fluorescence analysis is a fast and sensitive method, and has great potential application in trace detection of environmental toxins. However, many important environmental toxins are non-fluorescent substances, and it is still a challenge to construct a fluorescence detection method for non-fluorescent substances. Here, by means of charge transfer effect and smart molecular imprinting technology, we report a sensitive indirect fluorescent sensing mechanism (IFSM) and microcystin (MC-RR) is selected as a model target. A molecular imprinted thin film is immobilized on the surface of zinc ferrite nanoparticles (ZnFe_2_O_4_ NPs) by using arginine, a dummy fragment of MC-RR. By implementation of IFSM on the paper-based microfluidic chip, a versatile platform for the quantitative assay of MC-RR is developed at trace level (the limit of detection of 0.43 μg/L and time of 20 min) in real water samples without any pretreatment. Importantly, the proposed IFSM can be easily modified and extended for the wide variety of species which lack direct interaction with the fluorescent substrate. This work offers the potential possibility to meet the requirements for the on-site analysis and may explore potential applications of molecularly imprinted fluorescent sensors.

## Abstract (web summary)

Fluorescence analysis is a fast and sensitive method for the trace detection of environmental toxins, but it remains challenging to develop a fluorescence method for detecting nonfluorescent toxins. Here, the authors report an indirect fluorescent sensing strategy for the rapid, selective and sensitive detection of the non-fluorescent microcystin as a model target.

## Introduction

Fluorescent nanosensors have attracted great attention because of simplicity, high sensitivity, and throughput for chemo/bio assay and have become an important research direction of sensors and analytical chemistry^1^. Numerous types of fluorescent nanoprobe such as quantum dots^2^, nanoclusters^3^, nanodots^4^, polymer dots^5^, and so on, have been designed and used in fluorescent nanosensors. In general, sensing mechanism in such sensors is based on the direct interaction of target species with fluorescent nanoprobe to induce a decrease/enhancement of fluorescence emission and this change is tracked as the analysis response^6^. However, this sensing mechanism suffers from crucial problems, mainly including high susceptibility to the environmental variables, poor selectivity, and most importantly, inapplicability to target species that lack of interactions with fluorescent nanoprobes. The performance and selectivity of the fluorescent sensors can be promoted to a large extent by the functionalization (chemical modification) of fluorescent nanoprobes. In this regard, functional component(s) integrate to fluorescent nanoprobe and act as recognition moiety and bring target molecules to the fluorescent nanoprobe^7–10^.

Molecular imprinting technology refers to the formation of selective recognition sites in the polymer matrix with the memory of a template molecule^11^. Fluorescent nanoprobes can be incorporated into the molecular imprinted polymers (MIPs) with the aim of boosting sensitivity and selectivity of sensing scheme^12^. In MIPs-fluorescent sensors, recognition of target analyte through imprinted cavities is accompanied by the interaction between fluorescent nanoprobe and analyte, thus changing emission fluorescence intensity^13^. However, this strategy is limited to the target molecules that have direct interaction, such as static quenching, dynamic quenching, fluorescence resonance energy transfer (FRET), photo-induced electron transfer (PET), and inner filter effect (IFE) with fluorescent nanoprobe^13–17^. In other words, although most typical analyte can be selectively captured by the binding sites of MIPs, no obvious direct interaction by most analyte-nanoprobe is generated to construct a MIPs-nano fluorescent sensor. Specifically, competitive binding against fluorescent indicators (usually molecular fluorescent dyes) on MIPs is a possible indirect sensing method. The fluorescent indicators were replaced by target analytes through binding cavities competition. The fluorescence characteristic changes depending on how many fluorescent indicators are replaced^18^. For example, Hong et al. developed a competitive CdSe/ZnS quantum dot fluorescence assay based on micro-array-imprinted membranes for the determination of triazophos^19^. Furthermore, in order to take advantage of the nano-fluorescence probe in sensing mechanism, the fluorescent nano-materials not only act as fluorescent labels but also as a sensitive sensing source in indirect sensing system. Therefore, developing an alternative sensing mechanism is urgently demanded to surmount the mentioned dire obstacle and expand the application of fluorescent sensors.

Among the algal toxins, microcystins are produced by cyanobacteria, which are hepatotoxins, neurotoxins, and carcinogens. It can affect the heart, kidney, nervous system, and gastrointestinal tract in humans^20^. For this reason, the World Health Organization has set the threshold limit value of 1 μg/L for microcystin-LR. Canada sets a 20 μg/L guideline limit on total microcystins in recreational waters^21^. It has developed many methods for the detection of microcystins, like enzyme-linked immunosorbent assay^22^, liquid chromatography-tandem mass spectrometry^23^, high-performance liquid chromatography^24^, electrochemical assays^25^, and surface plasmon resonance bioassay^26^. However, these instruments and immunobioassays methods can be complex and expensive for detection of microcystins in surface waters. More recently, advanced fluorescent nanoprobes exhibited a large potential to establish effective methods for detection of microcystins^27–30^. However, most of the recognition elements of the constructed nanosensors are natural antibodies or their aptamers. Although natural antibodies and aptamer possess high affinity towards microcystins, they suffered from a number of major disadvantages such as being easily denatured, sensitive to the environmental variable, insufficient reproducibility, and high cost^31^. At the same time, molecular imprinted of microcystins, epitope imprinting, and dummy template imprinting are also evolving^32,33^. Therefore, it is obvious if MIPs can replace with antigens and aptamers to assemble fluorescence sensors for detection of microcystins, it will be a huge reduction in cost and upgrading the user-friendly and durability of the resultant sensor.

The good sensing method needs to be implemented on a compatible platform. Microfluidic paper-based device (μPADs), as a low-cost and user-friendly paper-based platform alternative to traditional laboratory testing, has improved the accessibility of the environmental analysis device under simple conditions^34^. The softness and toughness of plant fiber paper materials lay the foundation for the user-friendly operation of paper-based devices^35,36^. Our group has studied the molecularly imprinted fluorescent nanosensing on the paper-based microfluidic platform in previous works^37–39^. On the basis of our research, and literature search, main drawbacks of the direct fluorescence sensing of target species has been well comprehended and tried to eliminate thoroughly.

In this work, we have designed IFSM by choosing MC-RR as the model analyte, since it cannot enhance/quench the fluorescence emission of QDs. MIP thin film wrapped ZnFe_2_O_4_ NPs (ZnFe_2_O_4_@MIP) applies as a simulated quencher, which combines with fluorescent CdTe QDs to fabricate a functional paper-based chip. In this mechanism, imprinted cavities of MIP not only act as selective binding sites for capturing MC-RR molecules, but also they are the exclusive pathway of electron transfer between ZnFe_2_O_4_ NPs and CdTe QDs. Therefore, recognition of MC-RR is associated with filling the imprinted cavities which indirectly caused a significant enhancement of the fluorescence intensity of the QDs. Besides, slidable-clip type paper chip is designed by taking the advantage of the flexibility of paper materials to achieve the simultaneous analysis of double detection sites on IFSM.

## Results and discussion

### Preparation and operation of PQ-ZnFe_2_O_4_@MIPs μSPAD

**Fig. 1:**
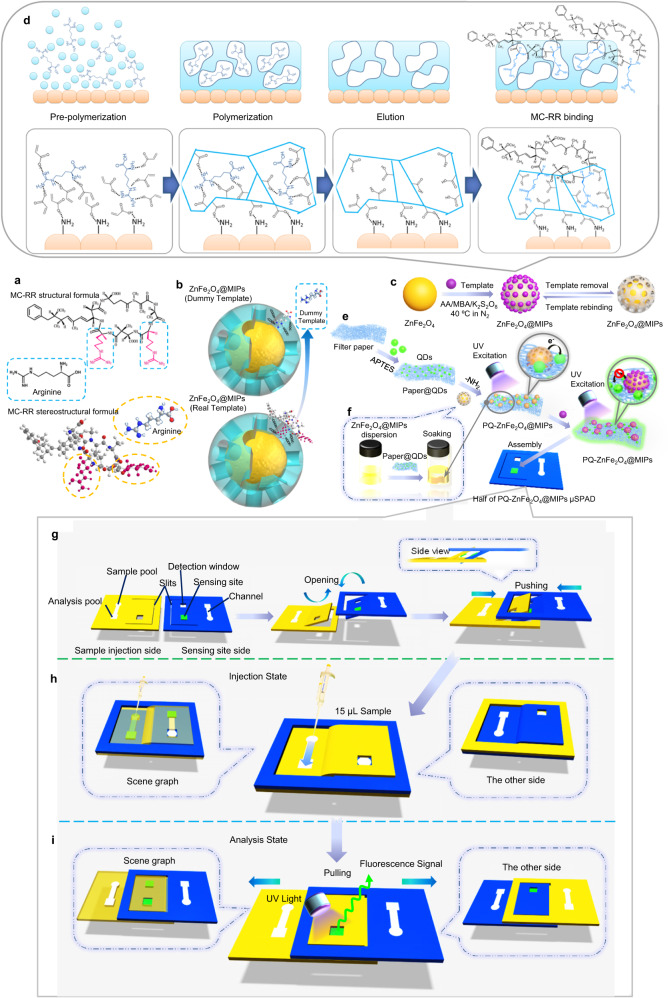
Schematic diagrams illustrating the structure of dummy fragment template imprinting technique and the preparation of PQ-ZnFe_2_O_4_@MIP μSPAD. The respective molecular structure of MC-RR and its arginine positions (**a**). The mechanism of the dummy fragment imprinting and rebinding MC-RR target (**b**). The synthetic method (**c**) and mechanism (**d**) of MIPs-wrapped ZnFe_2_O_4_ NPs. The manufacture of ZnFe_2_O_4_@MIPs modified paper-based fluorescence sensing site and corresponding sensing mechanism (**e**). Anchoring ZnFe_2_O_4_@MIPs on paper-based fluorescence substrate by soaking and shaker (**f**). **g** It displays various region functions of the μSPAD and assembly processes for the device. **h**, **i** Figure showed schematic diagram of operation process of sample injection (**h**) and fluorescence analysis (**i**) on the μSPAD.

MC-RR is highly toxic, harmful for manipulating, and very expensive for the process of synthesizing MIPs. Hence, the combination of the dummy molecular imprinting and fragment imprinting techniques is used to prepare MIPs film. As shown in Fig. 1a, arginine is a natural amino acid and has similar molecular shape and position of functional groups to two chains of MC-RR molecule. Strong hydrogen bonds can be generated between amino groups of arginine and the carboxyl group of functional monomers. Other than that, arginine is innocuous and much cheaper than MC-RR. Hence, using arginine qualify dummy fragment of MC-RR, that is preferable in terms of method expense and operational security. Just like antibodies recognizing specific groups on the surface of antigens, the synthesized MIPs can recognize arginine-like groups present in the MC-RR molecule (Fig. 1b). The synthesis procedure of thin MIPs layer on the surface of ZnFe_2_O_4_ NPs is schematically illustrated in Fig. 1c and the detailed mechanism is shown in Fig. 1d. During the pre-polymerization process, acrylic acid (AA) and arginine (Arg) were pre-bonded, and AA was aggregated on the surface of the ZnFe_2_O_4_ NPs particles due to the hydrogen bond between the amino group and the carboxyl group. After adding the *N*, *N*′-Methylenebisacrylamide (MBA, cross-linker) and the potassium persulfate (K_2_S_2_O_8_, initiator), under suitable conditions, the network-like gel state polymer embedded with arginine is formed and grows on the ZnFe_2_O_4_ NPs. Then, ZnFe_2_O_4_@MIPs is completed after breaking the hydrogen bond between the embedded Arg and the monomer in the polymer layer through the eluent, so that the templates could detach and MIP cavities are formed. The cavities of MIPs easily rebind the arginine on the MC-RR molecular fragment again. The paper-based fluorescence substrate is fabricated via amidation reaction between the amino groups of the amino-functionalized paper and the carboxyl groups of CdTe QDs (Fig. 1e). Subsequently, more amino groups are introduced to the paper-based fluorescence substrate by the sol-gel reaction to prepare paper@QDs-NH_2_. Finally, ZnFe_2_O_4_@MIPs is easily attached to paper-based fluorescence substrate by soaking and gentle oscillation due to the good adhesion and hydrophilic nature of imprinted polymer (Fig. 1f). In the absence of MC-RR, a hydrogen bonding would be produced between carboxyl groups in the MIP cavities and amino groups of the QDs, and the electron and energy transfer leads to fluorescence quenching of QDs. Due to the fluorescence resonance energy transfer (FRET) and photoinduced electron transfer (PET) theory, in the presence of the MC-RR, imprinted cavities of MIP layer are filled with MC-RR, and intermolecular forces are weakened between carboxyl groups (MIP). Meanwhile, QDs’ amino groups block the electron transfer, resulting in the recovery of QDs fluorescence intensity. Accordingly, the content of the template can be detected by the recovery rate of the QDs fluorescence intensity. Finally, PQ-ZnFe_2_O_4_@MIPs sensing site is pasted on the corresponding sensing site region of the chip to complete the fabrication of one of the PQ-ZnFe_2_O_4_@MIPs μSPADs and making another the same way (Fig. 1g).

The paper-based device consists of two identical paper chips, paired with each other. As shown in Fig. 1g and Supplementary Fig. 1, the two chips are flexible and inserted with each other through their own slits, which could simultaneously detect two sets of samples as a control experiment by µPADs. Such a creative design minimized the cost of external clips and avoided the tedious operation process of multi-layered origami paper chips and ensured the efficiency and convenience of detection. The sample injection (Fig. 1h) and analysis process (Fig. 1i) of the two detection sites can be achieved by facile operation (Supplementary Movie 1). More importantly, the detection sites do not interfere with each other because they are located on the different sides of the chip.

### Characterization and possible sensing mechanism of the PQ-ZnFe_2_O_4_@MIPs

**Fig. 2:**
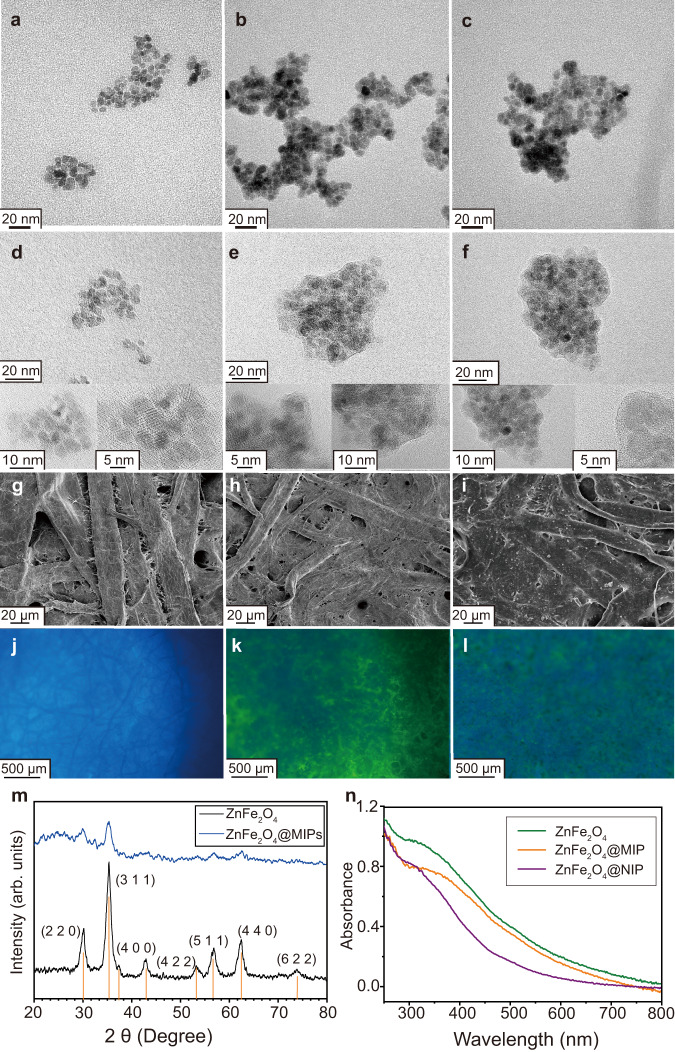
Morphology characterization of sensing material and systems. TEM images of ZnFe_2_O_4_ (**a**), ZnFe_2_O_4_@MIPs (**b**), and ZnFe_2_O_4_@NIPs (**c**). HRTEM images of ZnFe_2_O_4_ (**d**), ZnFe_2_O_4_@MIPs (**e**), and ZnFe_2_O_4_@NIPs (**f**). SEM images of bare paper (**g**), paper@QDs (**h**), PQ-ZnFe_2_O_4_@MIPs (**i**), corresponding fluorescence microscope images (**j**–**l**), and XRD pattern of the as-synthesized ZnFe_2_O_4_ and ZnFe_2_O_4_@MIPs (**m**). The vertical line at the bottom corresponds to the standard XRD pattern of ZnFe_2_O_4_ (JCPDS No. 22–1012). Absorption spectra of ZnFe_2_O_4_, ZnFe_2_O_4_@MIPs, and ZnFe_2_O_4_@MIPs, respectively (**n**). Reproducibility of the HRTEM, SEM, and fluorescence microscope image was verified by repeating three measurements.

The size and shape of ZnFe_2_O_4_ NPs and ZnFe_2_O_4_@MIPs were studied by the TEM and HRTEM. Figure 2s demonstrates the synthesized ZnFe_2_O_4_ materials are composed of well-dispersed spherical particles with an average diameter of 5–10 nm (The statistical data distribution as Supplementary Fig. 2A). After coating ZnFe_2_O_4_ NPs with MIPs layer (Fig. 2b), moderate change in the TEM image of ZnFe_2_O_4_@MIPs compared with bare ZnFe_2_O_4_ NPs is observed that is due to the thinness of the polymer layer. The MIP layer is clearer under HRTEM, shown in Fig. 2e, and its thickness is ~2–3 nm (The statistical data distribution as Supplementary Fig. 2B). Figure 2d, f show the TEM and HRTEM images of ZnFe_2_O_4_@NIP, respectively. The thickness and morphology of the non-imprinted polymer layer on the surface of ZnFe_2_O_4_ are the same as those of ZnFe_2_O_4_@MIP, and there is no obvious difference. The filter paper, paper@QDs, and PQ-ZnFe_2_O_4_@MIPs were characterized by SEM and fluorescence microscope as well. As seen in Fig. 2g, the filter paper is composed of interlaced fibers. In Fig. 2h, i, the observed dense materials cover distributed evenly on paper fibers are grafted QDs and ZnFe_2_O_4_@MIPs particles which successfully anchored. Fluorescence microscope images reveal the feasibility of fluorescence quenching on the paper-based platform (Fig. 2j–l).

The typical XRD pattern of the ZnFe_2_O_4_ NPs and ZnFe_2_O_4_@MIPs samples was also performed and the corresponding spectrum was illustrated in Fig. 2m. Most of the diffraction peaks could be completely matched with the standard data file of bulk cubic spinel-structured ZnFe_2_O_4_ (JCPDS 22–1012). In addition, successful immobilization of amorphous MIPs film on the ZnFe_2_O_4_ surface could be confirmed since an obvious amorphous peak was observed at 25° in the XRD pattern of the ZnFe_2_O_4_@MIPs. The differences in UV-Vis spectra ZnFe_2_O_4_ NPs and ZnFe_2_O_4_@MIPs were the other evidence of the successful coating of imprinted materials. As displayed in Fig. 2n, the unmodified ZnFe_2_O_4_ NPs had the strongest absorbance spectrum ranging from 300 nm to 600 nm. Thanks to diffuse reflection of light through polymer on the face of ZnFe_2_O_4_ NPs, the absorbance intensity of ZnFe_2_O_4_@MIPs and ZnFe_2_O_4_@NIPs were lower than that of unmodified ZnFe_2_O_4_ nanoparticles^40^. There were vacant cavities in the polymer layer of ZnFe_2_O_4_@MIPs that formed by the removal of the template. So, ZnFe_2_O_4_@MIPs absorbed light energy more easily than ZnFe_2_O_4_@NIPs, and its absorbance intensity was higher consequently. Supplementary Figure 3 indicated the FT-IR spectra of ZnFe_2_O_4_ and ZnFe_2_O_4_@MIPs. The characteristic bonds at 545 and 551 cm^−1^ observed in both of materials could be assigned to the Fe-O stretching vibration. In the ZnFe_2_O_4_@MIPs spectra, the stretching vibration of the C-H at 2930 and 2847 cm^−1^, C = O stretching at 1632, C-O stretching at 1394 and 1031 cm^−1^, and N-H stretching at 1535 cm^−1^ emerge. Thermogravimetric analysis (TGA) results demonstrated the PQ-ZnFe_2_O_4_@MIPs was stable below 300 °C and the weight percentage of immobilized QDs and ZnFe_2_O_4_@MIPs on the paper-based sensing substrate was estimated to be 3.5% (Supplementary Fig. 4).

**Fig. 3:**
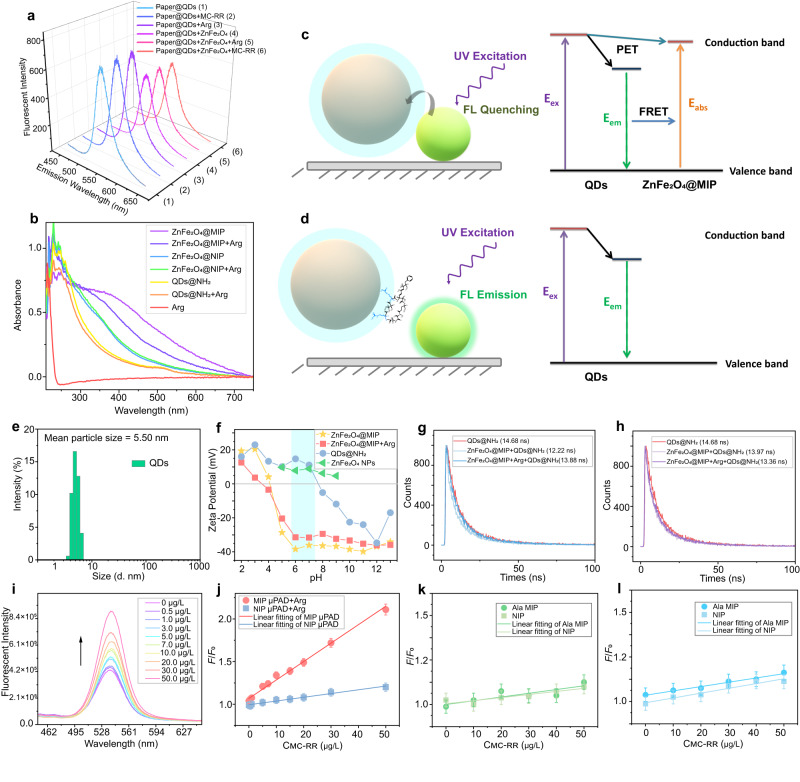
Sensing mechanism research data and schematic diagram. The effect of template (Arg) and MC-RR (50 μg/L of Arg and MC-RR) on imprinted-free sensing system (**a**). UV-Vis absorption spectra of different mixed systems (**b**). Schematic diagram of fluorescence quenching (**c**) and recovery (**d**) mechanism and corresponding system energy level and electron transition. Hydrated particle size distribution of CdTe QDs (mean particle size 5.502 nm) (**e**). Zeta potential distribution of ZnFe_2_O_4_@MIPs, ZnFe_2_O_4_@MIPs rebinding Arg, QDs-NH_2_, and ZnFe_2_O_4_ NPs under different pH conditions (**f**). Fluorescence lifetime spectrum of ZnFe_2_O_4_@MIPs system (**g**) and ZnFe_2_O_4_@NIPs system (**h**). Fluorescence spectrum (**i**) and linear diagram (**j**) of the detection effect of the PQ-ZnFe_2_O_4_@MIPs μSPAD on the detection of template molecule Arg (*n* = 4 independent experiments). Linear diagram of the detection effect of the PQ-ZnFe_2_O_4_@MIPs μSPAD on the detection of MC-RR, after replacing the template molecules with alanine (*n* = 4 independent experiments) (**k**) and lysine (**l**) in the synthesis process of ZnFe_2_O_4_@MIPs (*n* = 4 independent experiments). The error bars represent the standard deviation for relative parallel experiments.

The possible indirect fluorescent sensing mechanism (IFSM) was explored after characterizing the micro-topography and spectroscopy of the material involved. First, as shown in Fig. 3a, we used ZnFe_2_O_4_ NPs without polymer coating to prepare the sensing system. The sensing system did not respond at all after the addition of arginine. Similarly, arginine does not enhance the fluorescence emission of CdTe QDs. This led us to believe that the polymer layer coated on ZnFe_2_O_4_ NPs was the mediator that triggered the sensing. Based on previous results, ZnFe_2_O_4_ NPs showed a broad absorption spectrum in the UV-visible range (350–700 nm) because of charge-transfer transitions in this mixed-valence compound. This wavelength range coincides with the excitation wavelength and fluorescence emission wavelength of CdTe QDs, which creates conditions for the establishment of the inner-filter effect (IFE) and fluorescence resonance energy transfer. As expected, excitation and emission lights would be strongly absorbed by ZnFe_2_O_4_ NPs in the system involved ZnFe_2_O_4_ NPs and QDs, because of an IFE, thus attenuating the fluorescence intensity of the QDs. The IFE is possibly responsible for the slight asymmetric shape of the fluorescence spectrum peak of QDs in this sensing system^41^. However, FRET and photoinduced electron transfer are also possible mechanisms that cannot be ignored. After modification of ZnFe_2_O_4_ NPs with MIPs, because of the FRET and PET between ZnFe_2_O_4_ @MIPs and QDs, the fluorescence was quenched. As shown in Fig. 3b, we comprehensively investigated the absorption spectra of different hybrid systems. In the range of 350–700 nm, the absorbance of ZnFe_2_O_4_@MIPs is stronger than that of ZnFe_2_O_4_@NIPs, due to the vacant cavities. In addition, we found that when Arg was present, the absorbance after MIP adsorbed Arg was significantly lower than that without Arg, and this phenomenon is because of the rebinding of templates in cavities. According to Zeta potential distribution (shown in Fig. 3f), the charges on the QDs-NH_2_ and ZnFe_2_O_4_@MIPs surface were 11.1 mV and −31.6 mV in aqueous solution at pH 7, respectively. The zeta potential of ZnFe_2_O_4_ NPs was 8.86 mV, and the negative zeta potential attributed to the carboxyl groups on the surface of MIPs. It is most likely to generate electrostatic interaction between ZnFe_2_O_4_@MIP nanoparticles and positively charged amino-modified QDs through hydrogen bonding. As shown in Fig. 3c, the electrons in the excited state of the QDs could transfer to the conduction band of ZnFe_2_O_4_ NPs in the process of returning to the valence band, causing the fluorescence quenching. As illustrated in Fig. 3e, and hydrated particle size distribution of CdTe QDs was 5.50 nm (Mean particle size). According to previous spectroscopic and electrochemical studies, the conduction band energy level (Ecb) of the CdTe QDs (3.5 nm determined by HRTEM) (−1.6 V vs NHE) is more negative than the redox potential of the Zn^2+^/Zn^0^ (−0.76 V vs NHE), Fe^3+^/Fe^2+^ and Fe^2+^/Fe^0^ couples (−0.77 V and −0.44 V vs NHE). The free energy change of the electron transfer from the excited CdTe QDs to the active sites of Zn/Fe is thermodynamically favorable^42,43^. Moreover, according to the study of fluorescence lifetime (Fig. 3g, h), the existence of dynamic quenching of fluorescence resonance energy transfer is also indicated. The fluorescence lifetime of CdTe QDs was significantly decreased by ZnFe_2_O_4_@MIPs, and rebounded after the emergence of arginine. However, no similar phenomenon appeared in the ZnFe_2_O_4_@NIP system. The recognition and sensing process for MC-RR by the prepared PQ-ZnFe_2_O_4_@MIPs is schematically shown in Fig. 3d. By the recognition event in MIPs film, the electrostatic attraction between ZnFe_2_O_4_@MIPs and the primary amino groups on the surface of the QDs can be blocked. As a result, the fluorescence intensity of QDs is recovered at a certain degree, but it could not be restored to its original intensity owing to the existence of the inner-filter effect. Thus, MC-RR can be fluorescently detected by blocking electron transfer and FRET between QDs and ZnFe_2_O_4_ through the recognition of MIPs film. Finally, the quenching amount can be calculated by Stem-Volmer equation fitting^44^.

1 $$({F}_{0}\,/F)-1={K}_{{{{{{\rm{SV}}}}}}}\,{C}_{{{{{{\rm{M}}}}}}}$$

Where *F*_0_ and *F* represent the fluorescence intensity in the absence and presence of the template, respectively; *C*_M_ is the target concentration; and *K*_SV_ is the quenching constant for the quencher. The ratio of *K*_SV,MIPs_ to *K*_SV,NIPs_ is defined as the imprinting factor (IF), and (*F*_0_/*F*)−1 is defined as the quenching amount.

By setting a series of dummy templates (Arg) with concentration gradients, the fluorescence signal of the IFSM on PQ-ZnFe_2_O_4_@MIPs μSPAD recovered linearly, and the NIP group showed a good control effect, which verified the successful preparation of molecular imprinting and the method is in line with the expected principle (Fig. 3I, j). Comparing the effects of the dummy template (Arg), alanine (Fig. 3k), and lysine (Fig. 3l) during the synthesis of MIP layer, the detection effect of alanine and lysine of the final MC-RR were not ideal. This indicates that molecular imprinting synthesized by Arg as a dummy template molecule has certain structural advantages in recognizing MC-RR.

### Optimization of PQ-ZnFe_2_O_4_@MIPs μSPAD for detection of MC-RR

The optimization of relevant preparation and operation variables plays a vital role in the operational performance of the PQ-ZnFe_2_O_4_@MIPs μSPAD. In this regard, different effective factors, including synthesis conditions, buffer environment, dosage of sensing material, equilibration time, and elution time were investigated and optimized. Considering the very high cost of microcystins, it is hard to use for the MIPs template synthesis. Therefore, the arginine was used instead of MC-RR for all condition optimization experiments except for the investigation of detection time.

The amount of template molecule, functional monomer, and cross-linker has a great influence on the imprinting efficiency and selectivity of final MIPs. Different amounts of reagents were tested and results were present in Supplementary Fig. 5. The thickness of the MIPs film has a substantial impact on the efficiency of the IFSM. The quenching performance between ZnFe_2_O_4_ and QDs and sensing effect could be extremely suppressed if the thickness of the surface MIPs layer was beyond suitable value. The thickness of the MIPs layer and the stability of the MIPs film were accurately adjusted by using the optimum amount of cross-linker. According to the experimental results, 50 mg of arginine, 35 μL of AA, and 55 mg of MBA were chosen as optimum values for the preparation of ZnFe_2_O_4_@MIPs.

**Fig. 4:**
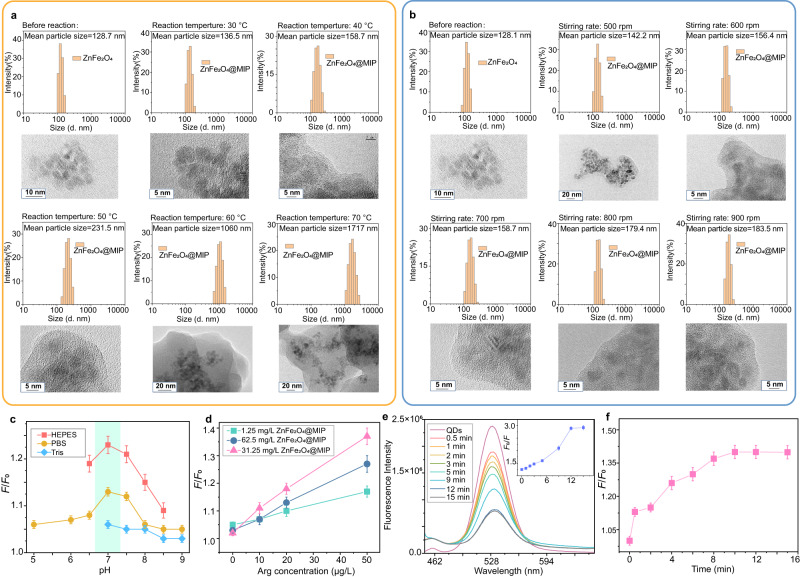
Conditional optimization results. The hydrated particle size distribution and high-definition TEM images of the synthesized ZnFe_2_O_4_@MIPs at different reaction temperatures (**a**) and different reaction stirring speeds (**b**). Optimized results of buffer system and pH value (10 μg/L of Arg, *n* = 3 independent experiments) (**c**). Optimization of concentration of ZnFe_2_O_4_@MIPs solution (3 min of equilibration time, *n* = 3 independent experiments) (**d**). The fluorescence quenching effect of incubation time of paper@QDs in ZnFe_2_O_4_@MIPs dispersion (**e**). Optimization of equilibration time after sample injection (20 μg/L of MC-RR, *n* = 4 independent experiments) (**f**). The error bars represent the standard deviation for relative parallel experiments.

The synthesis reaction temperature and stirring speed may affect the synthesis of the MIPs layer. Different temperatures (range of 30–70 °C) of reaction were investigated and optimized. As shown in Fig. 4a, the investigation results were characterized by hydrated particle size distribution and HRTEM. When the reaction temperature was 30 °C, the polymer layer formed on the surface of the nanoparticles was so thin that it could not even cover completely. Reacted at 40 and 50 °C, uniformly coated and moderately thick polymer layers were formed well. On the other hand, as displayed in Fig. 4b, the effect of stirring speed on the reaction needed moderate conditions. If the stirring speed is too low (<500 rpm), the nanoparticles are prone to sedimentation and aggregation in the long-term continuous reaction. With the increase of reaction stirring speed, the polymer on the surface of nanoparticles was more uniform. According to the experimental results, the reaction temperature of 40 °C and the stirring rate of 700 rpm were selected as the synthesis conditions.

Optimum elution time is essential for the complete arginine molecules removal and formation of accessible binding sites on the surface of ZnFe_2_O_4_ NPs. The effect of elution time was studied by measuring the fluorescence intensity of the sensor and sensing effect. As displayed in Supplementary Fig. 6, elution time in the range of 6 − 48 h was tested while the eluent was replaced every 6 h. As the time of elution increased, the template molecules were removed from the polymer membrane and the specific cavities were generated. The excess time of elution process may damage the structure of the MIPs and deformation of MIP binding sites. Hence, 24 h of elution was selected as the optimum elution time.

The type of different buffer systems has an impact on the sensing efficiency. The buffer concentration was all 10 mM, which was very high relative to the target in order to create a stable buffer environment. As shown in Fig. 4c, among three tested buffer solutions, the performance of the sensing system in HEPES buffer media was optimal. The poor sensing efficiency of the sensor in the PBS buffer system may be due to the strong binding energy between the high-concentration phosphate and the arginine, which could interfere with the binding between MIPs and arginine. The molecule of Tris has strong functionality with amino groups that let Tris molecules adsorb on the imprinted layer and occupy the cavities and affect performance.

The concentration of ZnFe_2_O_4_@MIPs solution for soaking of paper@QDs was also optimized and the results are demonstrated in Fig. 4d. The fluorescence enhanced effect of the paper@QDs socked by 31.25 mg/L of ZnFe_2_O_4_@MIPs was remarkably better than that was immersed in 62.5 or 125 mg/L of ZnFe_2_O_4_@MIPs. Moreover, the incubation time of paper@QDs in ZnFe_2_O_4_@MIPs solution was also examined. As indicated in Fig. 4e, a significant large-scale fluorescence quenching occurred immediately after the paper was immersed in the solution within 0.5 min. Subsequently, the fluorescence of the paper continued to quench until it remained stable after 12 min, which also verified the expected quenching mechanism. Thus, 15 min was chosen as the incubation time for paper@QDs in ZnFe_2_O_4_@MIPs solution.

To optimize the detection time, dynamic adsorption was also investigated over 12 min. As seen from Fig. 4f, the fluorescence enhancement effect increased within 12 min when the concentration of MC-RR was 20 μg/L, after 12 min the adsorption curve reached the plateau. Therefore, 12 min was chosen as the equilibrium time for further experiments.

### Analysis performance of PQ-ZnFe_2_O_4_@MIPs μSPAD

**Fig. 5:**
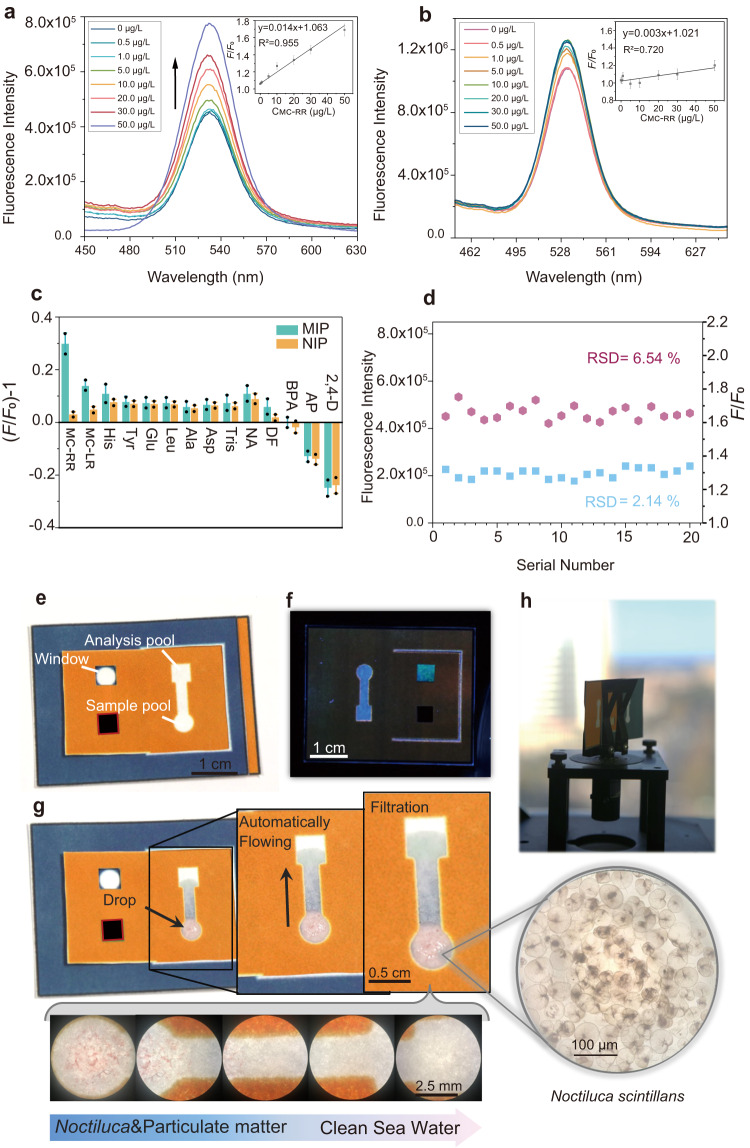
The performance of PQ- ZnFe_2_O_4_@MIPs μSPADs. Fluorescence spectrum responses diagrams of PQ- ZnFe_2_O_4_@MIPs μSPAD (**a**) and PQ- ZnFe_2_O_4_@NIPs μSPAD (**b**) for detection of MC-RR, and internal illustrations were calibration curves and linear equation (MC-RR concentration was 0, 0.5, 1, 5, 10, 20, 30, 50 μg/L respectively). (*n* = 4 independent experiments) **c** Selectivity of PQ- ZnFe_2_O_4_@MIPs μSPAD for MC-RR standard solutions (20 μg/L) of different interfering compounds. (*n* = 4 independent experiments) **d** Fluorescence spectrum peak intensity values (hexagonal shape) and sensing results (square shape) of twenty PQ-ZnFe_2_O_4_@MIPs μSPADs. **e** Photograph of the PQ-ZnFe_2_O_4_@MIPs μSPAD in sunlight (**e**) and UV light (365 nm) (**f**). The process of sample flowing through the chip channel and microscope images of *noctiluca scintillans* (×400) and hydrophilic channel of the device after sample injection (×100) (**g**). The device is placed on the testing platform of fluorescence spectrometer (**h**). The error bars represent the standard deviation for relative parallel experiments.

The sensitivity and performance of the paper-based device were examined by analyzing MC-RR standard aqueous solutions at various concentrations. The spectra in Fig. 5a revealed the fluorescence intensity of the sensing site enhanced with the increasing MC-RR concentration. The change in fluorescence intensity versus concentration of MC-RR has a linear response within the range of 0.5−50 μg/L with the regression equation of *y* = 0.014 x + 1.063 and correlation coefficient (*R*^2^) of 0.955 (inset Fig. 5a). In this case, according to the S/N > 3, by the formula 3σ/S (*σ* = 0.002008, and *S* = 0.014), the proposed paper-based device yielded a low limit of detection (LOD) of 0.43 μg/L. Besides, the NIP device was also used for the detection of MC-RR, and corresponding fluorescence spectra were present in Fig. 5b. The fluorescence recovery of NIP is negligible and the linear regression curve is inferior (the inset image of Fig. 5b), which further corroborates the blocking of electron transfer exclusively pertinent to the selective capturing of MC-RR by imprinted cavities. In addition, the initial fluorescence intensity of NIP is higher than that of MIP, proving the rationality of previously predicted QDs quenching and sensing mechanism.

To evaluate the selectivity of the PQ-ZnFe_2_O_4_@MIPs μSPAD, thirteen different potential interfering compounds including microcystins-LR (MC-LR), histidine (His), tyrosine (Tyr), glutamic acid (Glu), leucine (Leu), alanine (Ala), aspartic acid (Asp), tris(hydroxymethyl)aminoethane (Tris), niclosamide (NA), Dinotefuran (DF), bisphenol A (BPA), 2, 4-dichlorophenoxyacetic acid (2, 4-D) and acetamiprid (AP) were detected by the device. As displayed in Fig. 5c, the analytical response for other species exhibited no apparent fluorescence enhancement compared with MC-RR, while AP and 2, 4-D quench the fluorescence of QDs. The selectivity can be a result of the synergistic participation of functionality and spatial shape in the recognition of MC-RR. In addition, saline ions do not interfere with the sensing (Supplementary Fig. 7). These promising results proved the high selectivity and anti-interference ability of PQ- ZnFe_2_O_4_@MIPs μSPAD. Furthermore, the stability of the device was explored by testing the stability of fluorescence intensity and testing results of the 20 different devices (Fig. 5d) RSD values were as low as 6.54% and 2.14% that is strong evidence to affirm excellent stability of the proposed device. The temperature of the water sample at normal natural temperature (4–35 °C) did not affect the sensing results (Supplementary Fig. 8).

The appearance of μSPAD and the related sample process is demonstrated intuitively in Fig. 5e. The top layer contains a hydrophilic channel, sample pool, analysis pool, and window hole, and the sensing site emits weak fluorescence under the UV irradiation of 365 nm (Fig. 5e, f). Seawater sample contains high concentration of coexisting interfering species such as particulate matter and *Noctiluca scintillans*. The hydrophilic channel can transport the sample from the sample pool to the analysis pool by paper capillary wicking forces after dropping the sample. Interestingly, the microorganism and impurities can be filtered effectively by the water diversion channel (Fig. 5g). After the recognition reaction, the sensing sites are simply pulled to the window and performed a spectral analysis with a fluorescence spectrometer (Fig. 5h).

### Practical applications in real water samples

To examine the practical utility of the PQ-ZnFe_2_O_4_@MIPs μSPAD, four seawater, and lake water samples were collected from the coastal zone of the Yellow Sea (Yantai, China) and Yantai Sanyuanhu Lake. Only the seawater samples were diluted three times with HEPES buffer of pH 7. All tested samples were not found of MC-RR. Subsequently, a spiked recovery test at three concentration levels 1.0, 5.0, and 10.0 μg/L was carried out by the standard addition method and considered as a criterion of accuracy. The related analytical results are represented in Table 1. The recoveries of the samples obtained range from 92.1% to 109.8% and RSDs are from 4.41% to 6.77%, respectively.

**Table 1:** The results of application of the PQ-ZnFe_2_O_4_@MIPs μSPAD in real water

Sample		MC-RR spiked concentration	MC-RR detected results	Recovery ± RSDs
		(μg/L)	(μg/L)	%
Sea water	1	0	–	–
	2	5.00	5.49	109.8 ± 5.36
	3	10.00	10.55	105.5 ± 4.41
	4	15.00	13.82	92.1 ± 6.77
Sanyuanhu Lake water	1	0	–	–
	2	5.00	5.12	102.4 ± 4.28
	3	10.00	10.33	103.3 ± 4.14
	4	15.00	15.41	102.7 ± 5.23

Note: the natural pH of Sanyuan Lake water is 7.0. The seawater samples were diluted three times with HEPES buffer of pH 7.0.

(*n* = 5)

In order to further verify the performance of the proposed method, we used our method to detect MC-RR concentrations at seven locations in northern Taihu Lake, Jiangsu province, China (Supplementary Fig. 9). As shown in Supplementary Table 1 and Supplementary Fig. 10, by comparing the results obtained by liquid chromatography-mass spectrometry and our proposed method, it showed that the detection results of MC-RR by the two methods were consistent. The advantage of our method is that no sample pretreatment process is required, and the entire detection process is <20 min. And the detection process of liquid chromatography-mass spectrometry requires much more time due to the tedious pretreatment process. This demonstrates the good performance and potential application of our method on rapid on-site detection.

### Method performance comparison

The performance of the paper-based device for the detection of MC-RR is compared with the previously reported methods, as listed in Supplementary Table 2^45–48^. Most of the fluorescent assay systems for the detection of MC-RR require the intervention of aptamers and antibodies to use as a selective receptor. However, aptamers suffer from main drawbacks including the low success rate of synthesis and screening of aptamers condition, high cost, and instability in complex water samples. In addition, antibodies are very expensive, easily denatured, sensitive to the environmental variable, and possess insufficient reproducibility. PQ-ZnFe_2_O_4_@MIPs μSPAD method reduces the synthesis cost and promotes the applicability of fluorescence sensing in real water. Although the LODs of some previous methods are lower than the current method, the proposed sensing platform is convenient as practical applicability, since complex water samples can accurately be analyzed without consuming any sample pretreatment. Therefore, the developed PQ-ZnFe_2_O_4_@MIPs μSPAD method with simplicity, cheapness, validity, selectivity, user-friendliness, and a high potential for daily on-site assay proves to be an ideal candidate for MC-RR determination.

In summary, a universal indirect fluorescent sensing strategy is developed for rapid, selective, and sensitive detection of the non-fluorescent microcystin. The indirect fluorescent sensing mechanism is based on the combination of molecular imprinting technology and intramolecular charge transfer and is implemented on a slidable-clip type paper-based microfluidic chip. The function of IFSM relies on blocking the imprinted cavities of MIPs by recognition reaction which is the exclusive pathway of electron transfer between ZnFe_2_O_4_ NPs and QDs. By virtue of the good practical applicability of the designed μSPAD as well as the sensitivity and selectivity of IFSM, MC-RR as a model target can be quantitatively determined at a trace level in complicated water samples without any sample preparation.

It is expected that the development of an indirect fluorescent sensing mechanism will broaden the applicability of MIPs-fluorescence sensors, and can be extended for the target species that lack intrinsic fluorescence or they are inert to the fluorescent properties of fluorescent materials. It should be noted that in the proposed indirect fluorescent sensing mechanism, strong interaction between the target molecule and the imprinted cavity is necessary to achieve a more sensitive sensing effect. Therefore, how to synthesize molecularly imprinted coating materials with higher affinity needs to be further investigated. In addition, improving the anti-interference capability of the sensing system is also a key target for future research.

## Methods

### Materials and instruments

FeCl_3_·6H_2_O, ascorbic acid, sodium borohydride (NaBH_4_), *N*,*N*′-Methylenebisacrylamide (MBA), cadmium chloride (2CdCl_2_·5H_2_O), 4-(2-Hydroxyethyl)−1-piperazineethanesulfonic acid (HEPES), Tris(hydroxymethyl)aminoethane (Tris), *N*-hydroxysuccinimide (NHS), and 3-Ethylcarbodiimide hydrochloride (EDC) were purchased from the Aladdin Chemical Reagent Co. Ltd. 3-Aminopropyltriethoxysilane (APTES), thioglycollic acid (TGA), and 2-*N*-morpholinoethanesulfonic acid (MES) were purchased from Sigma–Aldrich (Shanghai, China). Zinc oxide (ZnO), tellurium powder, hydrazine hydrate, acetic acid, acrylic acid (AA), l-Arginine (Arg), potassium persulfate (K_2_S_2_O_8_), methanol, Na_2_HPO_4_, NaH_2_PO_4_ and HCL (0.02 M) were obtained from Sinopharm Chemical Reagent Co., Ltd (China). All reagents were of analytical grade and used as received. Ultrapure water (18.2 MΩ specific resistance) was produced by a Pall Cascada laboratory water system (Millipore, Bedford, MA, USA). The Whatman No.1 chromatography filter paper was supplied by GE Company (Shanghai, China).

Fluorescence spectra were obtained using a spectrofluorometer (Fluoromax-4, HORIBA): excitation light was set at 396 nm and the emission spectra were recorded from 450 to 650 nm for QDs. A fluorescence microscope (IX51, Olympus) equipped with a CCD camera was used to observe the fluorescence microscopy image on a glass slide. The purity of materials was studied by X-ray diffractometer (Shimadzu-7000X, Tokyo, Japan) with Cu Kα radiation. Infrared spectra were obtained from Fourier transform infrared spectrometer (Nicolet iS50, Thermo Scientific). UV/vis absorption spectra were recorded on a spectrophotometer (NanoDrop2000/2000C, Thermo Scientific). Thermogravimetric analysis (TGA) used a Mettler 5MP/PF7548/MET/400 W thermal analyzer in air atmosphere and the heating rate was 10 °C/min (Mettler Toledo, Switzerland). The shape, size, and morphological evaluation were recorded by a transmission electron microscope (TEM, JEM-1230, operating at 100 kV) and scanning electron microscope (SEM, Hitachi S-4800, Japan). Zeta potential measurements were performed on a Malvern Zetasizer Nano-ZS90 (ZEN3590, UK). The paper-based chip was fabricated using a commercial solid-wax printer (XEROX Phaser 8560DN).

### Synthesis of MIPs-wrapped ZnFe_2_O_4_ NPs (ZnFe_2_O_4_@MIPs)

Firstly, ZnFe_2_O_4_ NPs were synthesized by hydrothermal method according to a reported method^49^. 0.192 g of ZnO powder, 0.776 g FeCl_3_ and 0.424 g ascorbic acid were dispersed in 32 mL ultrapure water by ultrasonication for 30 s in a beaker, and subsequently, 8 mL of hydrazine hydrate was added to the mixture and stirred for 20 min. Afterwards, the mixture was transferred into a 50 mL Teflon-lined stainless steel autoclave, sealed and heated at 180 °C for 12 h, then cooled down naturally. The obtained products were centrifuged (12,320 g, centrifugal radius = 10 cm, 5 min) and washed with ultrapure water and absolute ethanol several times, and then dried at 60 °C in the air oven, and ready for use.

Secondly, the ZnFe_2_O_4_@MIPs was prepared via a facile free radical polymerization process similar to the reported procedures with some modifications^50^. Arg, AA, MBA, and potassium persulfate were used as dummy fragment template, functional monomer, cross linker, and initiator, respectively. Briefly, 5 mg ZnFe_2_O_4_ NPs, 50 mg of Arg, and 35 μL AA were mixed in 15 mL of ultrapure water, and the mixture was stirred for 120 min under the protection of nitrogen. Next, 55 mg of MBA was added to the above solution. After fully mixing for 15 min, 10 mg of potassium persulfate was added, and kept stirring at 40 °C under the protection of nitrogen stream overnight in the dark (~15 h). The obtained MIPs-coated ZnFe_2_O_4_ were centrifuged (12,320 g, centrifugal radius = 10 cm, 5 min) and washed with ethanol/acetic acid (9:1, v/v) to remove template molecules. Finally, the ZnFe_2_O_4_@MIPs were washed with ultrapure water three times and dispersed in 2 mL of ultrapure water for further use. As a control, the non-imprinted material (ZnFe_2_O_4_@NIPs) was prepared in the same manner but without adding a dummy fragment template Arg.

### Synthesis of amino-functionalized paper-based fluorescence substrate (paper@QDs-NH_2_)

The CdTe QDs were synthesized according to our previous study^37^. The freshly prepared NaHTe aqueous solution (using 40 mg of NaBH_4_ and 38.3 mg of tellurium powder) was injected into the cadmium precursor solution (92.4 mg of Cd(NO_3_)_2_·4H_2_O, 63 μL of TGA, and 75 mL of distilled water) under stirring and protection of nitrogen. The mixture was adjusted to pH 9.0, followed by heating the system to 96 °C and refluxing for 1 h. The synthesized TGA-modified CdTe QDs exhibited strong bright green fluorescence at 530−545 nm.

The synthesis of the fluorescent filter paper substrate method was improved on the basis of our previous research^37^. The Whatman No. 1 filter paper was cut into 15 mm × 15 mm and dipped in 1 mol/L NaOH solution. The filter paper and NaOH solution were transferred and sealed into a Teflon-lined stainless-steel autoclave, heated at 120 °C for 2 h. After cooling down to room temperature, paper chips were washed with ultrapure water several times for further use. The filter paper was immersed in 20 mL of ethanol/water (50%, v/v) solution and 200 μL of APTES was added to the above mixture and oscillated with an oscillator for 8 h. After washing with ultrapure water, the paper substrate was temporarily stored in the Petri dish and waiting for the next step. In all, 6 mL of 20 mg/mL EDC was added into 8 mL CdTe quantum dots and kept under magnetic stirring for about 5 min. Then 6 mL of 10 mg/mL NHS was added. A total of 20 mL above mixture was slowly added to four pieces of paper and reacted by oscillation at room temperature in the dark for 8 h to obtain paper chips grafted quantum dots (paper@QDs). The product was rinsed three times with ultrapure water. Finally, the paper@QDs was immersed in 20 mL of ethanol/water (50%, v/v) solution that contained 10 μL APTES for 5 h for amino functionalization of grafted at QDs on the paper fiber. The obtained paper@QDs-NH_2_ was stored at 4 °C in the dark for the subsequent experiments.

### Manufacture of ZnFe_2_O_4_@MIPs modified paper-based fluorescence sensing site (PQ-ZnFe_2_O_4_@MIPs)

The paper@QDs-NH_2_ was soaked and made gentle oscillation in the ZnFe_2_O_4_@MIPs solution for 15 min on the oscillator. Then, the paper chips were taken out and dried at room temperature and cut into 5 mm × 5 mm size to get the final paper-based fluorescence sensing site (PQ-ZnFe_2_O_4_@MIPs). The paper-based sensing sites were all stored in the dark, dry, and sealed before use.

### Design and fabrication of slidable-clip type paper-based device (PQ-ZnFe_2_O_4_@MIPs μSPAD)

The chip was designed with drawing software (Adobe Illustrator). The paper-based device consists of two identical paper chips that are paired with each other (as shown in Supplementary Fig. 1). Taking one of them as an example, the dimensions of the chip obtained are 50 mm × 40 mm. The sample pool is 5 mm in diameter, and the channel length between the sample pool and the square analysis pool (5 × 5 mm^2^) is 8 mm. On the side of the hydrophobic channel, there are two square regions for the sensing sites (5 mm × 5 mm) and two holes used for cutting the window respectively (displayed in Supplementary Fig. 1H). The C-type slits (the total length is 66 mm) are designed as sliding track and crossed entrance, with a distance of 6 mm from the long side of the chip and 3 mm from the short side of the chip.

The chip was directly printed onto the filter paper (Whatman chromatography No. 1 paper, GE) by a wax printer (XEROX Phaser 8560DN). Then let the wax penetrate through the paper completely to form a hydrophobic barrier by keeping the paper in an oven at 150 °C for 30 s according to the previous reports^37^. Prepared paper-based QDs sensing substrate (PQ-ZnFe_2_O_4_@MIPs) was affixed to the chip sensing site 1 and site 2 (green areas). Finally, a pair of paper-based chip was interspersed with each other for assembly. The slidable-clip type paper-based device (PQ-ZnFe_2_O_4_@MIPs μSPAD) was constructed.

### Usage of PQ-ZnFe_2_O_4_@MIPs μSPAD

The convenient and user-friendly usage of PQ-ZnFe_2_O_4_@MIPs μSPAD for fluorescence assay procedures is detailed below. The two chips were pushed in the opposite direction until the analysis pool of one chip overlap with the sensing site of another chip. Meanwhile, the circular sample pool of one chip was exposed through the square window of another chip. 15 μL of the sample was dropped on one of the sample pools. After 12 min of equilibrium, the two chips were pulled out in the opposite direction until the sensing site of one chip was exposed through the square window of another chip. The reaction was terminated and the sensing site was analyzed by fluorescence spectrometer. Two different sensing sites could be detected by turning the front and back sides of the paper-based device, in order to avoid interference between the two sites.

### Reporting summary

Further information on research design is available in the Nature Portfolio Reporting Summary linked to this article.

### Supplementary information

Supplementary information

Reporting Summary

Description of Additional Supplementary Files

Reporting Summary

Supplementary Movie 1

### Source data

Source Data

## Data Availability

The authors declare that the data supporting the findings of this study are available within the article and supplementary information. Additional datasets related to this work are available from the corresponding author upon request. Source data are provided in this paper. Source data are provided with this paper.

## References

[1] Zhou JJ, Chizhik AI, Chu S, Jin DY (2020). Single-particle spectroscopy for functional nanomaterials. Nature.

[2] Medintz IL, Uyeda HT, Goldman ER, Mattoussi H (2005). Quantum dot bioconjugates for imaging, labelling and sensing. Nat. Mater..

[3] Algar WR (2021). Photoluminescent nanoparticles for chemical and biological analysis and imaging. Chem. Rev..

[4] Baker SN, Baker GA (2010). Luminescent carbon nanodots: emergent nanolights. Angew. Chem. -Int. Ed..

[5] Wu CF, Chiu DT (2013). Highly fluorescent semiconducting polymer dots for biology and medicine. Angew. Chem. -Int. Ed..

[6] Li MX, Chen T, Gooding JJ, Liu JQ (2019). Review of carbon and graphene quantum dots for sensing. ACS Sens..

[7] Iovino F, Merkl P, Spyrogianni A, Henriques-Normark B, Sotiriou GA (2020). Silica-coated phosphorescent nanoprobes for selective cell targeting and dynamic bioimaging of pathogen-host cell interactions. Chem. Commun..

[8] Li Z, Liang T, Lv SW, Zhuang QG, Liu ZH (2015). A rationally designed upconversion nanoprobe for in vivo detection of hydroxyl radical. J. Am. Chem. Soc..

[9] Chan WCW, Nie SM (1998). Quantum dot bioconjugates for ultrasensitive nonisotopic detection. Science.

[10] Melnychuk N, Klymchenko AS (2018). DNA-functionalized dye-loaded polymeric nanoparticles: Ultrabright FRET platform for amplified detection of nucleic acids. J. Am. Chem. Soc..

[11] Chen LX, Wang XY, Lu WH, Wu XQ, Li JH (2016). Molecular imprinting: perspectives and applications. Chem. Soc. Rev..

[12] Haupt K, Mosbach K (2000). Molecularly imprinted polymers and their use in biomimetic sensors. Chem. Rev..

[13] Ivanova-Mitseva PK (2012). Cubic molecularly imprinted polymer nanoparticles with a fluorescent core. Angew. Chem. -Int. Ed..

[14] Descalzo AB, Somoza C, Moreno-Bondi MC, Orellana G (2013). Luminescent core-shell imprinted nanoparticles engineered for targeted forster resonance energy transfer-based sensing. Anal. Chem..

[15] Cheubong C (2021). Molecularly imprinted polymer nanogel-based fluorescence sensing of pork contamination in halal meat extracts. Biosens. Bioelectron..

[16] Ton XA, Acha V, Haupt K, Bernadette TSB (2012). Direct fluorimetric sensing of UV-excited analytes in biological and environmental samples using molecularly imprinted polymer nanoparticles and fluorescence polarization. Biosens. Bioelectron..

[17] Stringer RC, Gangopadhyay S, Grant SA (2010). Detection of nitroaromatic explosives using a fluorescent-labeled imprinted polymer. Anal. Chem..

[18] Piletsky SA (1997). Optical detection system for triazine based on molecularly-imprinted polymers. Anal. Lett..

[19] Hong S (2019). A Novel CdSe/ZnS quantum dots fluorescence assay based on molecularly imprinted sensitive membranes for determination of triazophos residues in cabbage and apple. Front. Chem..

[20] Basu A (2018). Assessment of hepatotoxic potential of cyanobacterial toxins using 3d in vitro model of adult human liver stem cells. Environ. Sci. Technol..

[21] Brophy MJ, Trueman BF, Park Y, Betts RA, Gagnon GA (2019). Fluorescence spectra predict microcystin-LR and disinfection byproduct formation potential in lake water. Environ. Sci. Technol..

[22] Fischer WJ (2001). Congener-independent immunoassay for microcystins and nodularins. Environ. Sci. Technol..

[23] Cadel-Six S (2014). Detection of free and covalently bound microcystins in different tissues (liver, intestines, gills, and muscles) of rainbow trout (Oncorhynchus mykiss) by liquid chromatography-tandem mass spectrometry: method characterization. Environ. Pollut..

[24] Zhang LF, Ping XF, Yang ZG (2004). Determination of microcystin-LR in surface water using high-performance liquid chromatography/tandem electrospray ionization mass detector. Talanta.

[25] Abnous K (2019). An ultrasensitive electrochemical sensing method for detection of microcystin-LR based on infinity-shaped DNA structure using double aptamer and terminal deoxynucleotidyl transferase. Biosens. Bioelectron..

[26] Sun XL (2013). Longitudinal surface plasmon resonance assay enhanced by magnetosomes for simultaneous detection of Pefloxacin and Microcystin-LR in seafoods. Biosens. Bioelectron..

[27] Yu HW, Kim IS, Niessner R, Knopp D (2012). Multiplex competitive microbead-based flow cytometric immunoassay using quantum dot fluorescent labels. Anal. Chim. Acta.

[28] Lv JJ, Zhao S, Wu SJ, Wang ZP (2017). Upconversion nanoparticles grafted molybdenum disulfide nanosheets platform for microcystin-LR sensing. Biosens. Bioelectron..

[29] Zhang YL (2019). Enzyme-free fluorescent detection of microcystin-LR using hairpin DNA-templated copper nanoclusters as signal indicator. Talanta.

[30] Lee EH, Son A (2019). Fluorescence resonance energy transfer based quantum dot-Aptasensor for the selective detection of microcystin-LR in eutrophic water. Chem. Eng. J..

[31] Van Dorst B (2010). Recent advances in recognition elements of food and environmental biosensors: a review. Biosens. Bioelectron..

[32] Matsui J, Fujiwara K, Takeuchi T (2000). Atrazine-selective polymers prepared by molecular imprinting of trialkylmelamines as dummy template species of atrazine. Anal. Chem..

[33] Yang KG (2019). Epitope imprinting technology: Progress, applications, and perspectives toward artificial antibodies. Adv. Mater..

[34] Salentijn GIJ, Grajewski M, Verpoorte E (2018). Reinventing (bio)chemical analysis with paper. Anal. Chem..

[35] Gong MM, Sinton D (2017). Turning the page: Advancing paper-based microfluidics for broad diagnostic application. Chem. Rev..

[36] Martinez AW, Phillips ST, Whitesides GM, Carrilho E (2010). Diagnostics for the developing world: microfluidic paper-based analytical devices. Anal. Chem..

[37] Li BW (2017). Quantum dot-based molecularly imprinted polymers on three-dimensional origami paper microfluidic chip for fluorescence detection of phycocyanin. ACS Sens..

[38] Qi J (2017). Three-dimensional paper-based microfluidic chip device for multiplexed fluorescence detection of Cu^2+^ and Hg^2+^ ions based on ion imprinting technology. Sens. Actuat. B-Chem..

[39] Qi J (2018). Rotational paper-based microfluidic-chip device for multiplexed and simultaneous fluorescence detection of phenolic pollutants based on a molecular-imprinting technique. Anal. Chem..

[40] Deng F, Li YX, Luo XB, Yang LX, Tu XM (2012). Preparation of conductive polypyrrole/TiO2 nanocomposite via surface molecular imprinting technique and its photocatalytic activity under simulated solar light irradiation. Colloid Surf. A..

[41] Kim J (2006). Magnetic fluorescent delivery vehicle using uniform mesoporous silica spheres embedded with monodisperse magnetic and semiconductor nanocrystals. J. Am. Chem. Soc..

[42] Jasieniak J, Califano M, Watkins SE (2011). Size-dependent valence and conduction band-edge energies of semiconductor nanocrystals. ACS Nano.

[43] Yue DT (2017). Sulfurated NiFe -based layered double hydroxides nanoparticles as efficient co-catalysts for photocatalytic hydrogen evolution using CdTe/CdS quantum dots. Appl. Catal. B-Environ..

[44] Stanisavljevic M, Krizkova S, Vaculovicova M, Kizek R, Adam V (2015). Quantum dots-fluorescence resonance energy transfer-based nanosensors and their application. Biosens. Bioelectron..

[45] Shi Y (2012). A graphene oxide based biosensor for microcystins detection by fluorescence resonance energy transfer. Biosens. Bioelectron..

[46] Wang FF (2015). Colorimetric detection of microcystin-LR based on disassembly of orient-aggregated gold nanoparticle dimers. Biosens. Bioelectron..

[47] Wu SJ, Li Q, Duan N, Ma HL, Wang ZP (2016). DNA aptamer selection and aptamer-based fluorometric displacement assay for the hepatotoxin microcystin-RR. Microchim. Acta.

[48] Taghdisi SM (2017). A novel fluorescent aptasensor for ultrasensitive detection of microcystin-LR based on single-walled carbon nanotubes and dapoxyl. Talanta.

[49] Zhang J (2015). ZnFe_2_O_4_ nanoparticles: synthesis, characterization, and enhanced gas sensing property for acetone. Sens. Actuat. B-Chem..

[50] Yu JL (2017). One-pot synthesis of a quantum dot-based molecular imprinting nanosensor for highly selective and sensitive fluorescence detection of 4-nitrophenol in environmental waters. Environ. -Sci. Nano.

