# Supplementary material for: Molecular imprinting-based indirect fluorescence detection strategy implemented on paper chip for non-fluorescent microcystin
Source: Nat Commun. 2023 Oct 17;14:6553. doi: 10.1038/s41467-023-42244-z (PMC10582162; doi:10.1038/s41467-023-42244-z)
Supplement: Supplementary file 1 — Supplementary information [file 41467_2023_42244_MOESM1_ESM.pdf]

Supplementary Information

Molecular Imprinting-Based Indirect Fluorescence Detection Strategy Implemented on Paper Chip For Non-Fluorescent Microcystin

Bowei Li[1,6], Ji Qi[1,6,*], Feng Liu[1], Rongfang Zhao[1,6], Maryam Arabi[1,6], Abbas Ostovan[1,6], Jinming Song[2,4,6*], Xiaoyan Wang[5], Zhiyang Zhang[1,6], Lingxin Chen[1,3,6*]

[1]CAS Key Laboratory of Coastal Environmental Processes and Ecological Remediation, Yantai Institute of Coastal Zone Research, Chinese Academy of Sciences, Yantai 264003, China

[2] CAS Key Laboratory of Marine Ecology and Environmental Sciences, Institute of Oceanology, Chinese Academy of Sciences, Qingdao 266071, China

[3] Laboratory for Marine Biology and Biotechnology, Pilot National Laboratory for Marine Science and Technology, Qingdao 266237, China

[4] Laboratory for Marine Ecology and Environmental Sciences, Qingdao National Laboratory for Marine Science and Technology, Qingdao 266237, China

[5] School of Pharmacy, Binzhou Medical University, Yantai 264003, China

[6] Center for Ocean Mega-Science, Chinese Academy of Sciences, Qingdao 266071, China

*E-mail: jqi@yic.ac.cn (J. Qi), jmsong@qdio.ac.cn (J. Song), lxchen@yic.ac.cn (L. Chen)

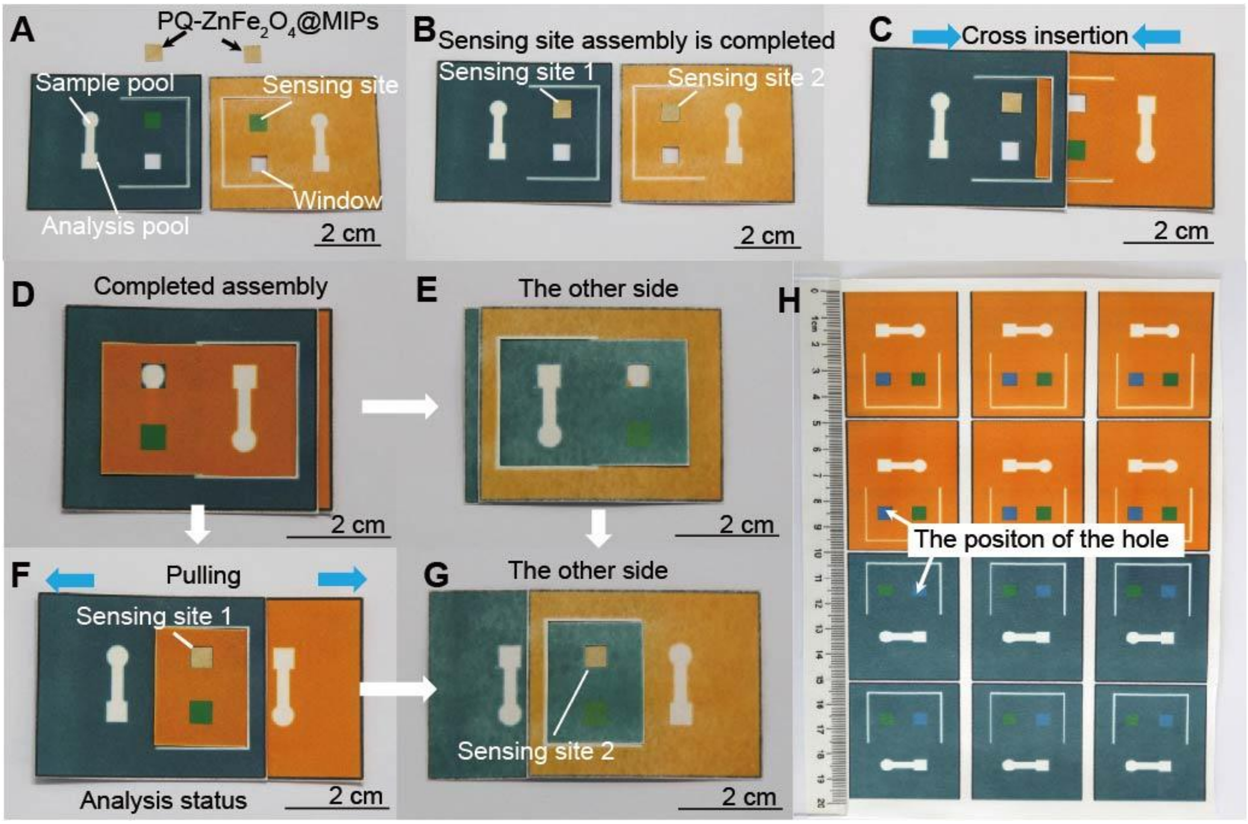

Supplementary Figure 1. (A) Photographs of the components of PQ-$ZnFe_2O_4$@MIPs μSPAD that included paper-based sensing substrate (PQ-$ZnFe_2O_4$@MIPs) and a pair of paper chip. (B) The sensor site is assembled. (C) The two parts of the chip were cross inserted into each other and combined. The top view (D) and bottom view (E) of the assembled PQ-$ZnFe_2O_4$@MIPs μSPAD. The top view (F) and bottom view (G) of the analysis status of the PQ-$ZnFe_2O_4$@MIPs μSPAD by pulling and the sensing sites were exposed through windows, respectively. (H) The paper chips printed in batches and their dimensions.

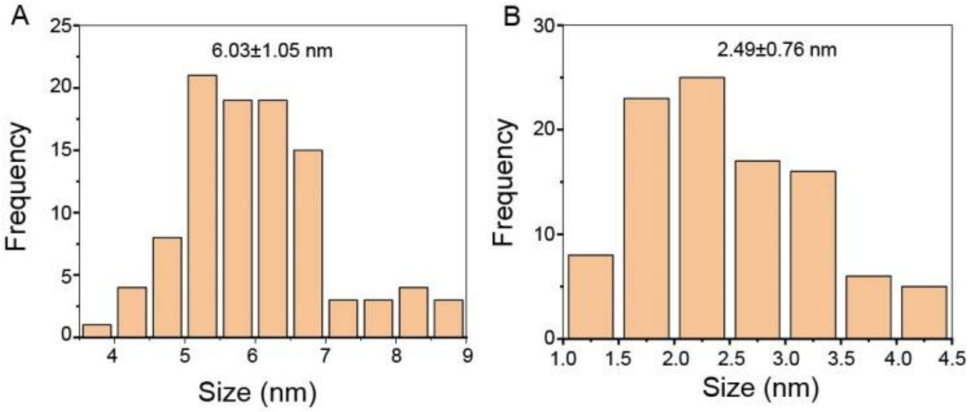

Supplementary Figure 2. (A) The statistical data distribution for size of 100 of $ZnFe_2O_4$ nanoparticles in the TEM image through descriptive statistics method. statistical data distribution for thickness of MIPs layer in the TEM image through descriptive statistics method ($P < 0.05$) .

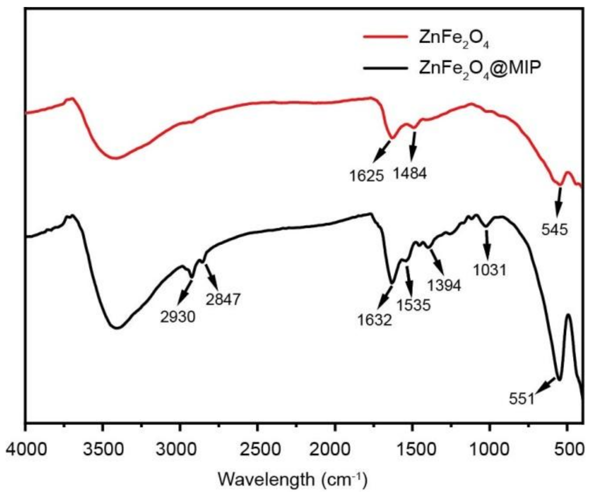

Supplementary Figure 3. Fourier transform infrared spectroscopy (FT-IR) of $ZnFe_2O_4$ and $ZnFe_2O_4$@MIP.

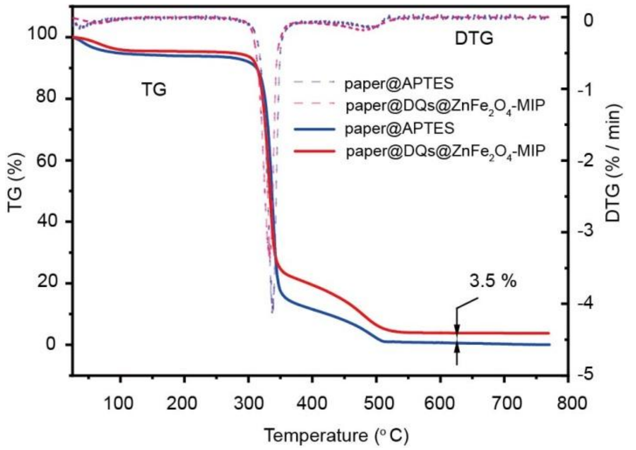

Supplementary Figure 4. Thermogravimetric analysis (TGA) curves of the PQ-$ZnFe_2O_4$@MIPs and paper@APTES, at a heating rate of 10 ^{o}C/min from room temperature to 800 ^{o}C under air atmosphere.

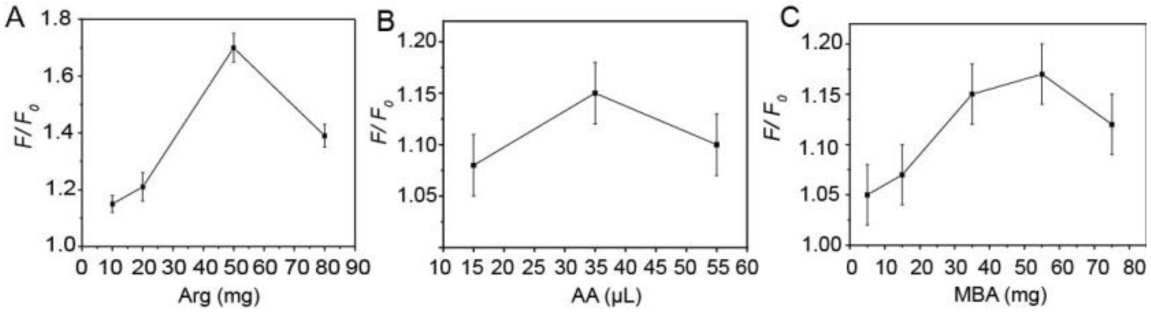

Supplementary Figure 5. Influences of the amount of of template (arginine) (A), function monomer (AA) (B) and cross-linking agent (MBA) (C). The investigation of template was completed in 50 μg/L of MC-RR (HEPES, 0.1 mol/L, pH 7.0, n=5) and the investigation of function monomer and cross-linking agent were completed in 5 μg/L of MC-RR (HEPES, 0.1 mol/L, pH 7.0, n=5 independent experiments, and the error bars represent the standard deviation.).

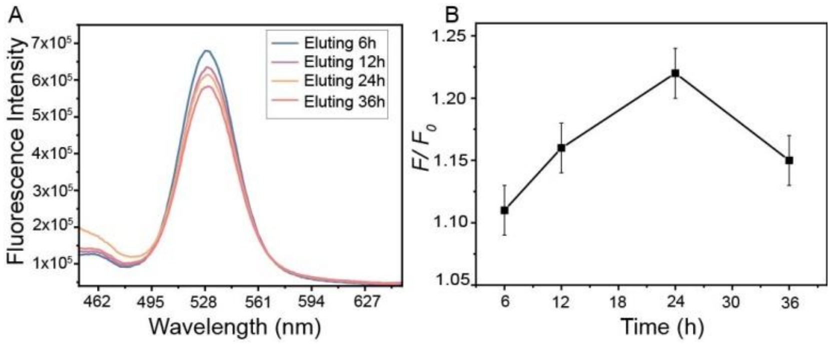

Supplementary Figure 6. The fluorescence spectra of PQ-$ZnFe_2O_4$@MIPs after eluting template for different times (6 h, 12 h, 24 h and 48 h) (A) and the sensing effect in 10 μg/L of MC-RR (HEPES, 0.1 mol/L, pH 7.0, n=5 independent experiments, and the error bars represent the standard deviation.) (B).

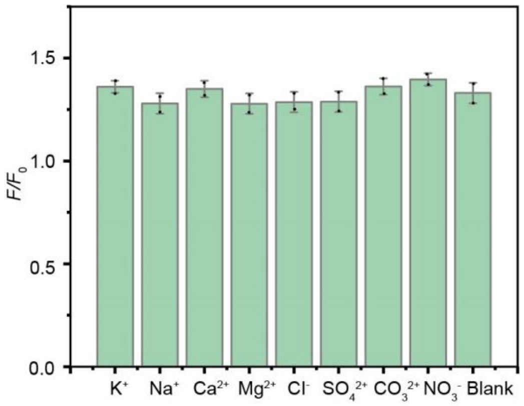

Supplementary Figure 7. The investigation of interference of saline ions (1 g/L of K^+, Na^+, Ca^{2+}, Mg^{2+}, Cl^-, SO_4^{2-}, CO_3^{2-}, NO_3^-, Blank, n=3 independent experiments, and the error bars represent the standard deviation.) for detection of MC-RR (20 μg/L) .

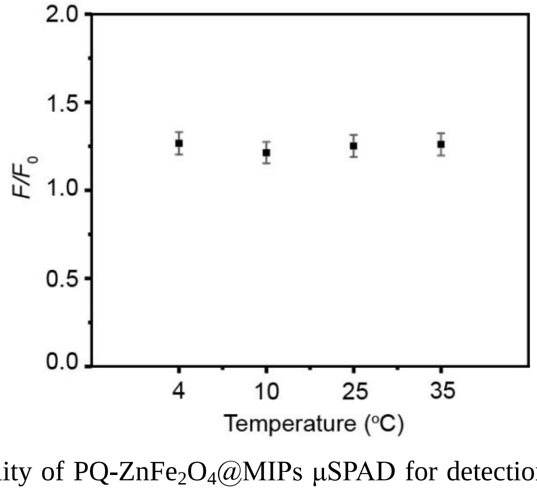

Supplementary Figure 8. Stability of PQ-$ZnFe_2O_4$@MIPs μSPAD for detection of samples (contain 20 μg/L of MC-RR) on different temperature (n=3 independent experiments, and the error bars represent the standard deviation.).

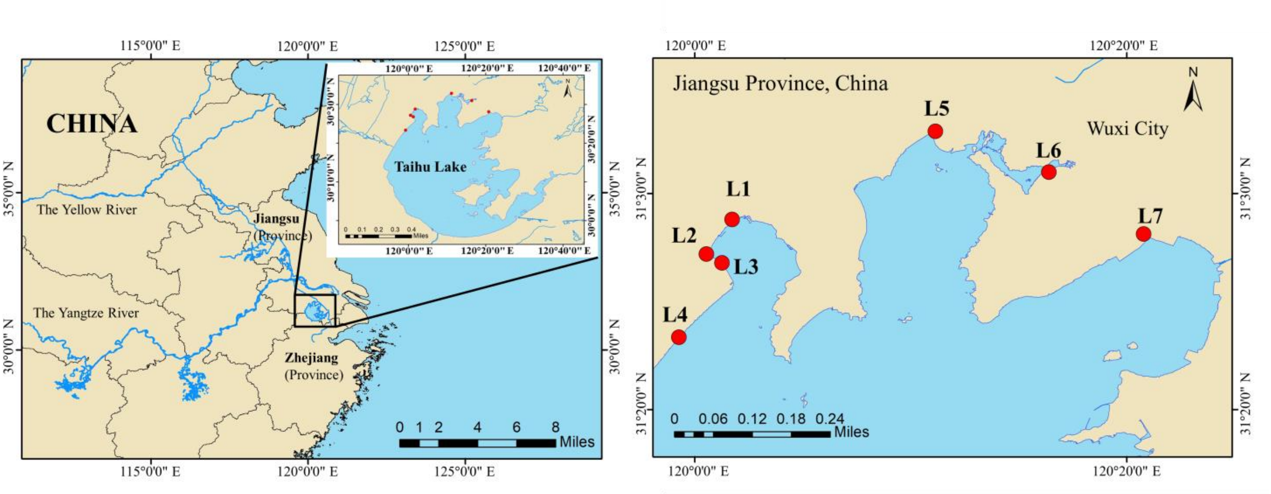

Supplementary Figure 9. Geographical location map of seven sampling locations (L1-L7) in Taihu Lake, Jiangsu, China. (Samples were collected at June, 3, 2023).

Supplementary Table 1. The results of MC-RR concentration of 7 samples from seven locations in the Taihu Lake detected by liquid chromatography-mass spectrometry (LC-MS) and this method.

Method	MC-RR (μg/L)						
	L1	L2	L3	L4	L5	L6	L7
**LC-MS**	0.435	0.422	0.319	3.208	0.345	0.092	Not found
**This method**	0.594	0.630	0.551	4.640	0.430	Not found	Not found

Method of LC-MS:

The samples were collected and stored in low temperature (<4 ℃) away from light. In order to guarantee the accuracy of the results, all the LC-MS analysis was carried out through third-party testing agency named “scientific compass company” in China .We sent the samples to their testing center through the service of ice pack insulation, and the subsequent extraction and LC-MS testing processes were all tested in accordance with the standardized procedures stipulated by the national standard.

Extraction: Wash and activate the HLB extraction cartridge (Oasis HLB 6cc (200 mg), purchased from Waters Corporation, U.S.A.) with 5 mL of methanol and 5 mL of double distilled pure water. A 1000 mL water sample was taken, adjusted to neutrality with HCl and NaOH, and then 10 mL of methanol was added. The flow rate of the water sample through the HLB extraction cartridge was 10 mL/min. After the water sample was enriched, the solid-phase extraction cartridge was dried with nitrogen. The enriched solid-phase extraction cartridge was then eluted with 15 mL methanol/0.2% formic acid mixed solution (v:v, 9:1), and the eluate was collected, dehydrated with anhydrous sodium sulfate, and concentrated.

Concentration: Concentrate the extraction eluate with a nitrogen blower, and accurately dilute to 1 mL with the mobile phase.

The liquid chromatography conditions are as follows:

Chromatographic column is C18, 50 mm × 2.1 mm (i.d.), 1.7 μm

Sample chamber temperature: 10 ℃. Column oven temperature: 40 ℃.

Injection volume: 10 μL

Mobile phase A: acetonitrile, mobile phase B: ultrapure water (0.2% formic acid)

Tandem mass spectrometry conditions:

Electrospray ionization (ESI) positive ion mode was used for detection, and the monitoring mode was multi-stage reaction monitoring mode (MRM). The injection voltage was 1.5 kV, the source temperature was 150 ℃, the desolvation temperature was 450 ℃, the flow rate was 800 L/h, and the cone gas flow rate was 150 L/h.

Using the mobile phase to dilute the standard solution of MC-RR to concentrations of 10.0, 20.0, 50.0, 100.0, 200.0 μg/L. Concentration from low to high sample injection detection, quantitative ion peak area-concentration plotted to obtain the standard curve regression equation. For the LC-MS calibration, the linear equation was F(x)=5185.31x+0, with correlation coefficient (R^2) 0.998. Through the testing, the peak area values of the blank samples were detected to be 83, 11, and 59, respectively. Therefore, the obtained standard deviation σ was 36.6. According to the S/N>3, the LOD was obtained by the formula 3σ/S (σ=36.6, and S=5185.3) was 0.02 μg/L. The limit of quantitation (LOQ) was 0.05 μg/L for LC-MS method. The samples to be tested were tested under the same conditions as those for drawing the standard curve, and the quantitative result data was substituted into the linear regression equation to obtain the concentration result.

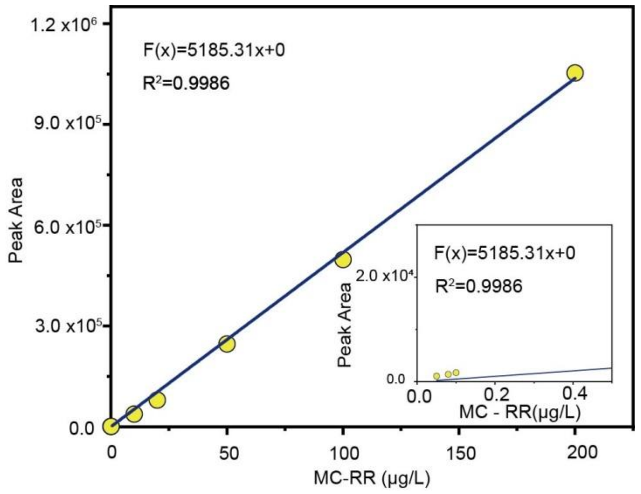

Supplementary Figure 10. The standard curve of LC-MS method for detection of MC-RR.

Supplementary Table 2. Performance comparison with other reported methods for microcystins testing.

System	Method and platform	Long-term preservation or not	Time taken for testing	Detection range	LOD (μg/L)	Supplementary Ref.
**Au–DNA–MCs complexes**	Fluorescence assay Glass slip	Not	180 min	0.5–2.5 μg/L	0.50	[1]
**AuNP dimers linked by aptamers**	Colorimetric assay Cuvettes	Not	65 min	0.1–248.8 μg/L	0.05	[2]
**Aptamer-based GO-SELEX**	Fluorescence PCR 96-well microplate	Not	140 min	0.20–2.5 μg/L	0.08	[3]
**SWNTs-based dapoxyl dye and aptamer-DAP-10**	Fluorescence assay Cuvettes	Not	75 min	0.4–1200 μg/L	0.14	[4]
**PQ-$ZnFe_2O_4$@MIPs μSPAD**	Fluorescence assay Paper-based chip	Yes	20 min	0.5–50 μg/L	0.43	This work

Supplementary References

[1] Y. Shi, J. Z. Wu, Y. J. Sun, Y. Zhang, Z. W. Wen, H. C. Dai, H. D. Wang, Z. Li, *Biosens. Bioelectron.* **2012**, *38*, 31-36.

[2] F. F. Wang, S. Z. Liu, M. X. Lin, X. Chen, S. R. Lin, X. Z. Du, H. Li, H. B. Ye, B. Qiu, Z. Y. Lin, L. H. Guo, G. N. Chen, *Biosens. Bioelectron.* **2015**, *68*, 475-480.

[3] S. J. Wu, Q. Li, N. Duan, H. L. Ma, Z. P. Wang, *Microchim. Acta* **2016**, *183*, 2555-2562.

[4] S. M. Taghdisi, N. M. Danesh, M. Ramezani, N. Ghows, S. A. M. Shaegh, K. Abnous, Talanta 2017, 166, 187-192.
