# Supplementary material for: Molecular imprinting-based indirect fluorescence detection strategy implemented on paper chip for non-fluorescent microcystin
Source: Nat Commun. 2023 Oct 17;14:6553. doi: 10.1038/s41467-023-42244-z (PMC10582162; doi:10.1038/s41467-023-42244-z)
Supplement: Supplementary file 2 — Reporting Summary [file 41467_2023_42244_MOESM2_ESM.pdf]

Corresponding author(s): Lingxin Chen

Last updated by author(s): Oct 3, 2023

Reporting Summary

Nature Portfolio wishes to improve the reproducibility of the work that we publish. This form provides structure for consistency and transparency in reporting. For further information on Nature Portfolio policies, see our Editorial Policies and the Editorial Policy Checklist.

Statistics

For all statistical analyses, confirm that the following items are present in the figure legend, table legend, main text, or Methods section.

n/a	Confirmed	
☐	☒	The exact sample size (n) for each experimental group/condition, given as a discrete number and unit of measurement
☒	☐	A statement on whether measurements were taken from distinct samples or whether the same sample was measured repeatedly
☒	☐	The statistical test(s) used AND whether they are one- or two-sided<br>*Only common tests should be described solely by name; describe more complex techniques in the Methods section.*
☒	☐	A description of all covariates tested
☒	☐	A description of any assumptions or corrections, such as tests of normality and adjustment for multiple comparisons
☐	☒	A full description of the statistical parameters including central tendency (e.g. means) or other basic estimates (e.g. regression coefficient) AND variation (e.g. standard deviation) or associated estimates of uncertainty (e.g. confidence intervals)
☒	☐	For null hypothesis testing, the test statistic (e.g. F, t, r) with confidence intervals, effect sizes, degrees of freedom and P value noted<br>*Give P values as exact values whenever suitable.*
☒	☐	For Bayesian analysis, information on the choice of priors and Markov chain Monte Carlo settings
☒	☐	For hierarchical and complex designs, identification of the appropriate level for tests and full reporting of outcomes
☒	☐	Estimates of effect sizes (e.g. Cohen's d, Pearson's r), indicating how they were calculated

Our web collection on statistics for biologists contains articles on many of the points above.

Software and code

Policy information about availability of computer code

Data collection	No software was used.
Data analysis	Origin software.

For manuscripts utilizing custom algorithms or software that are central to the research but not yet described in published literature, software must be made available to editors and reviewers. We strongly encourage code deposition in a community repository (e.g. GitHub). See the Nature Portfolio guidelines for submitting code & software for further information.

Data

Policy information about availability of data

All manuscripts must include a data availability statement. This statement should provide the following information, where applicable:

- Accession codes, unique identifiers, or web links for publicly available datasets
- A description of any restrictions on data availability
- For clinical datasets or third party data, please ensure that the statement adheres to our policy

The authors declare that the data supporting the findings of this study and they are available within the article and supplementary information. Additional datasets related to this work are available from the corresponding author upon request. Source data are provided in this paper.

Research involving human participants, their data, or biological material

Policy information about studies with human participants or human data. See also policy information about sex, gender (identity/presentation), and sexual orientation and race, ethnicity and racism.

Reporting on sex and gender	Our research does not address sex and gender descriptions.
Reporting on race, ethnicity, or other socially relevant groupings	Our research does not address race, ethnicity and other socially relevant groupings.
Population characteristics	Our research does not address population characteristics.
Recruitment	Our research does not address participants.
Ethics oversight	Our research does not address protocol.

Note that full information on the approval of the study protocol must also be provided in the manuscript.

Field-specific reporting

Please select the one below that is the best fit for your research. If you are not sure, read the appropriate sections before making your selection.

☐ Life sciences ☐ Behavioural & social sciences ☒ Ecological, evolutionary & environmental sciences

For a reference copy of the document with all sections, see nature.com/documents/nr-reporting-summary-flat.pdf

Ecological, evolutionary & environmental sciences study design

All studies must disclose on these points even when the disclosure is negative.

Study description	In order to verify the effectiveness of the method in the actual environmental water samples containing MC-RR, we collected water samples from 7 different locations in the northern part of Taihu Lake (Jiangsu province, China) on June 3, 2023. The detection results obtained by liquid chromatography-mass spectrometry and our proposed method. By liquid chromatography-mass spectrometry for detection, The samples were treated using solid-phase extraction. The number of experiment units were 7.
Research sample	In order to choose the time and location where blue-green algae are prone to outbreaks. The sample is a real water inTaihu Lake that affects the lives of urban populations. Water samples from 7 different locations in the northern part of Taihu Lake on June 3, 2023.
Sampling strategy	Each sample size is 1 L, which meets the needs of subsequent testing. Randomly select from the central part of the environmental water body at the designated location. Collect samples and store in low temperature (<4°C) away from light.
Data collection	By comparing the results obtained by liquid chromatography-mass spectrometry and our proposed method. Ji Qi recorded the test data with a pen and paper.
Timing and spatial scale	Water samples from 7 different locations (Supplementary Figure 9) in the northern part of Taihu Lake on June 3, 2023. All samples are collected in 12 h. The samples were collected and stored in low temperature (<4°C) away from light. We collected samples within 12 hours to minimize the impact of local weather on the samples.
Data exclusions	No data were excluded from the analyses.
Reproducibility	5 independent experiments were repeated on paper-based microfluidic chip for each sample. The reproducibility of the experimental findings was verified with actual samples.
Randomization	Disrupting the test order of samples in multiple tests ensures randomization.
Blinding	The type and serial number of the sample are not disclosed to the tester.

Did the study involve field work? ☐ Yes ☒ No

Reporting for specific materials, systems and methods

We require information from authors about some types of materials, experimental systems and methods used in many studies. Here, indicate whether each material, system or method listed is relevant to your study. If you are not sure if a list item applies to your research, read the appropriate section before selecting a response.

Materials & experimental systems

n/a	Involved in the study
☒	☐ Antibodies
☒	☐ Eukaryotic cell lines
☒	☐ Palaeontology and archaeology
☒	☐ Animals and other organisms
☒	☐ Clinical data
☒	☐ Dual use research of concern
☒	☐ Plants

Methods

n/a	Involved in the study
☒	☐ ChIP-seq
☒	☐ Flow cytometry
☒	☐ MRI-based neuroimaging
