# Supplementary material for: Molecular imprinting-based indirect fluorescence detection strategy implemented on paper chip for non-fluorescent microcystin
Source: Nat Commun. 2023 Oct 17;14:6553. doi: 10.1038/s41467-023-42244-z (PMC10582162; doi:10.1038/s41467-023-42244-z)
Supplement: Supplementary file 3 — Description of Additional Supplementary Files [file 41467_2023_42244_MOESM3_ESM.pdf]

Title: Supplementary Movie 1:
Description: The assembling paper-based microfluidic chip and its detection process for MC-RR
