# Supplementary figures and images for: Molecular imprinting-based indirect fluorescence detection strategy implemented on paper chip for non-fluorescent microcystin
Source: Nat Commun. 2023 Oct 17;14:6553. doi: 10.1038/s41467-023-42244-z (PMC10582162; doi:10.1038/s41467-023-42244-z)

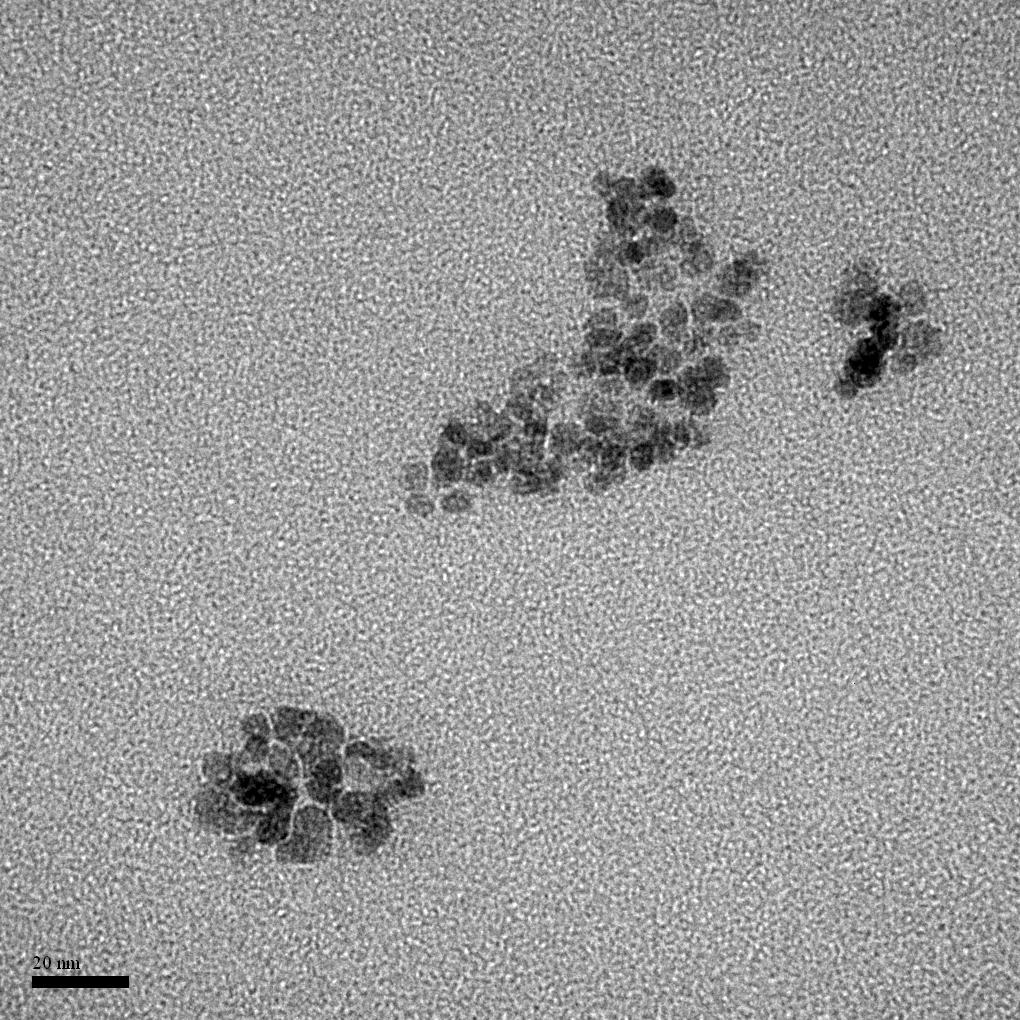

Supplement: Supplementary file 6 — Source Data [file 41467_2023_42244_MOESM6_ESM.zip › Original data-final/Figure 2 a-l/(a)/100000.0V600000X21847.jpg]

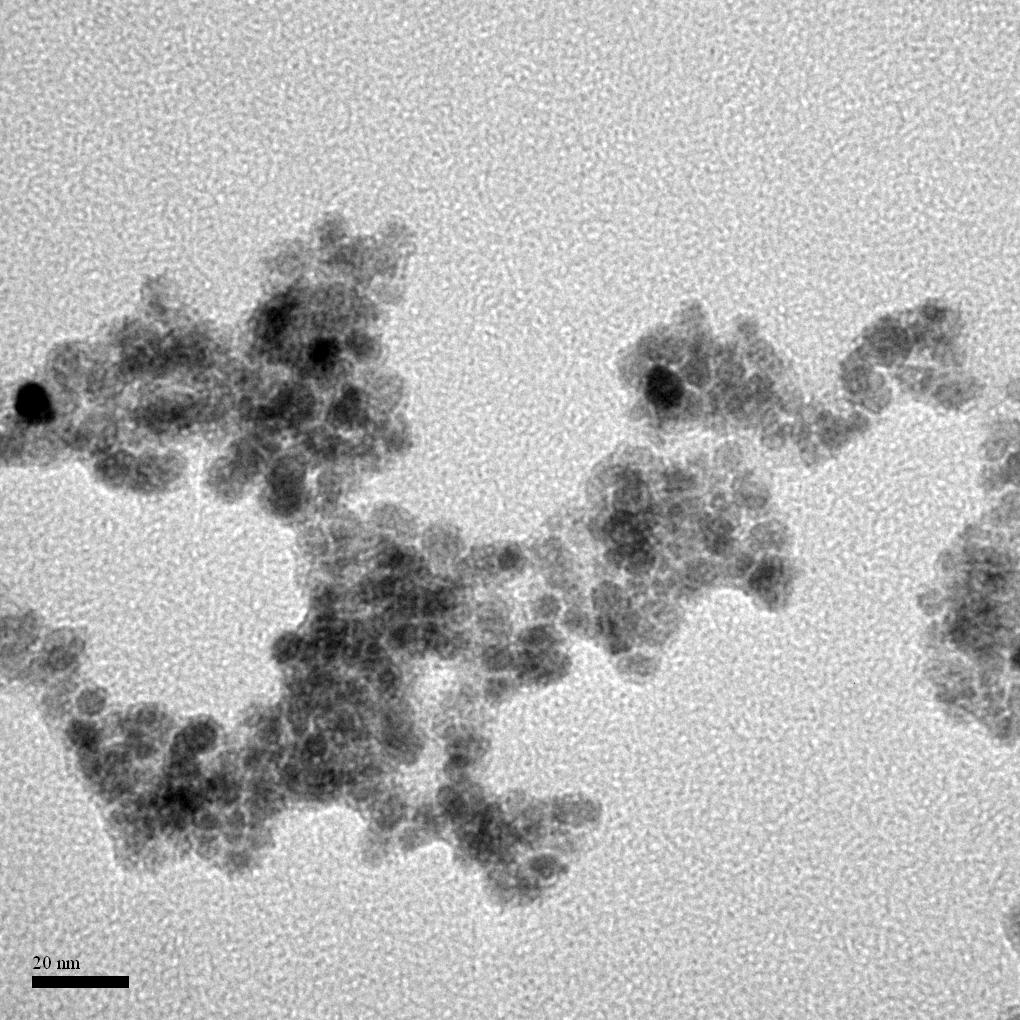

Supplement: Supplementary file 6 — Source Data [file 41467_2023_42244_MOESM6_ESM.zip › Original data-final/Figure 2 a-l/(b)/100000.0V600000X21869.jpg]

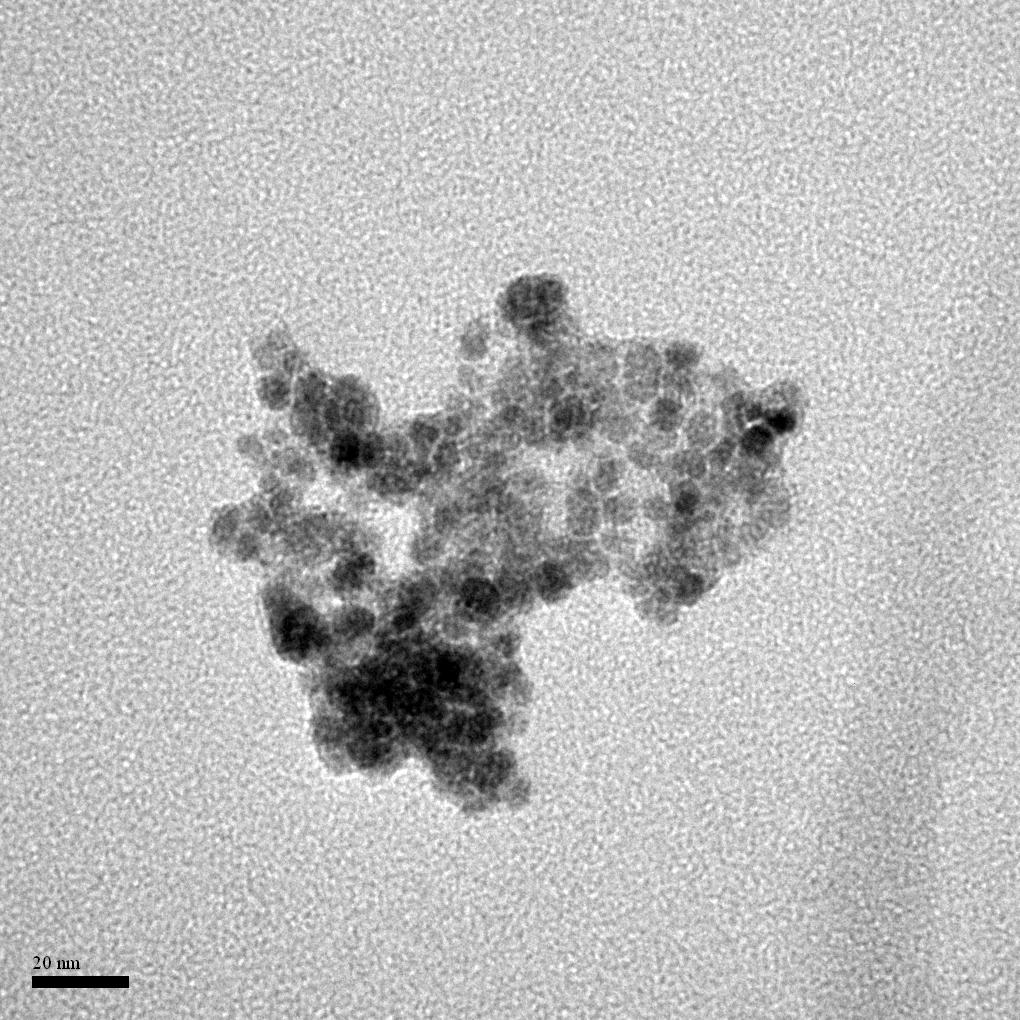

Supplement: Supplementary file 6 — Source Data [file 41467_2023_42244_MOESM6_ESM.zip › Original data-final/Figure 2 a-l/(c)/100000.0V600000X21865.jpg]

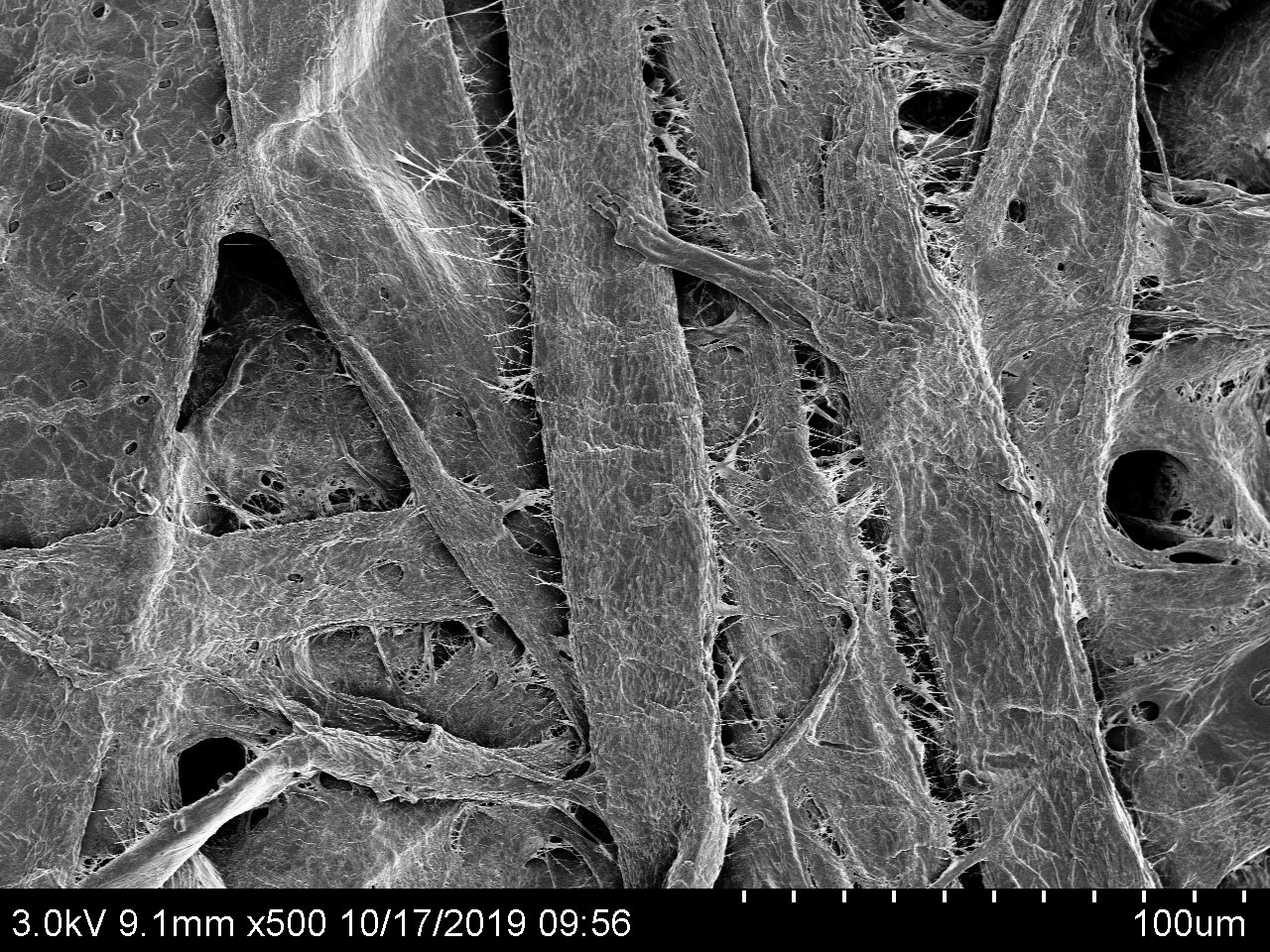

Supplement: Supplementary file 6 — Source Data [file 41467_2023_42244_MOESM6_ESM.zip › Original data-final/Figure 2 a-l/(g)/1_m001.jpg]

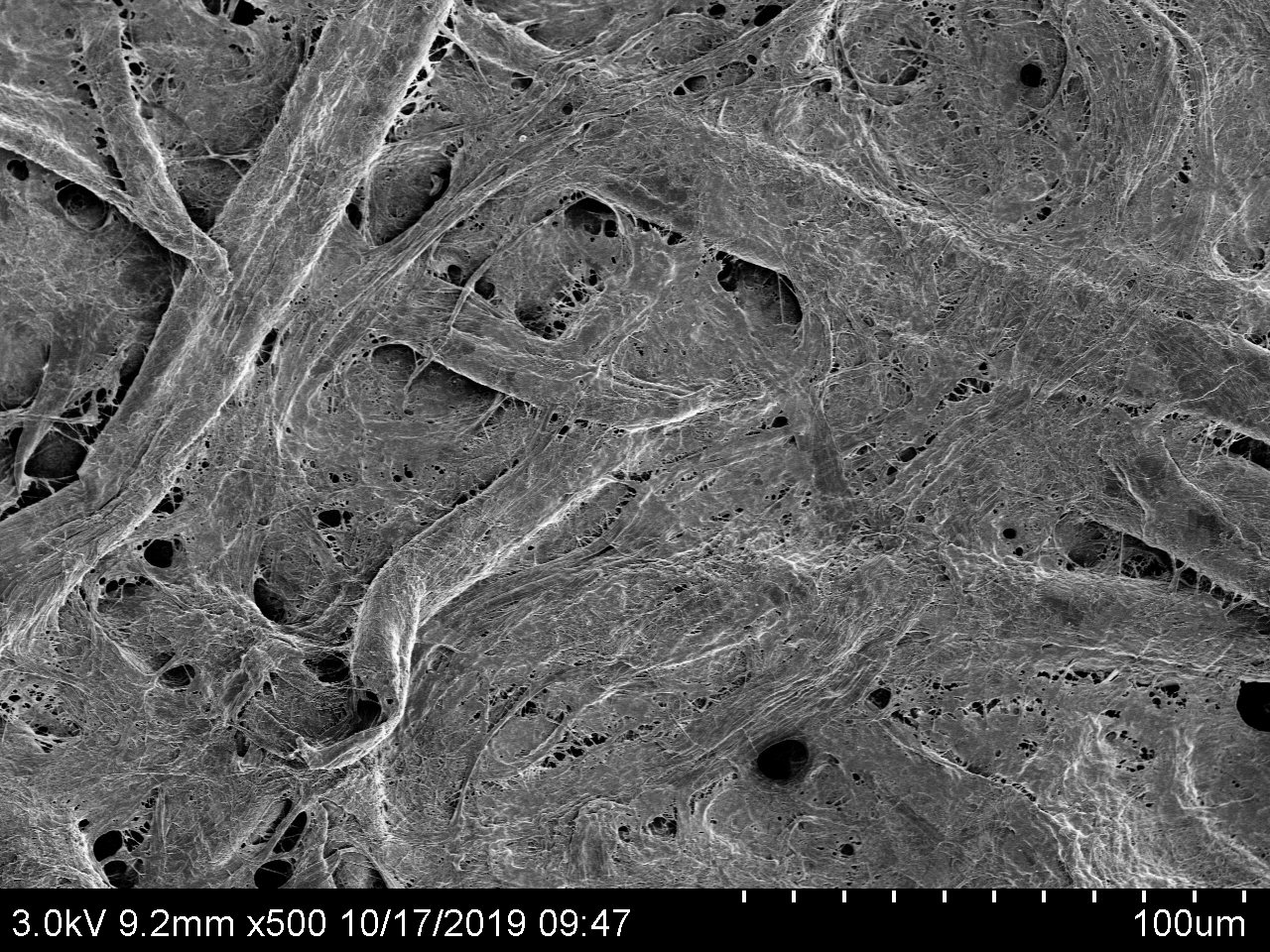

Supplement: Supplementary file 6 — Source Data [file 41467_2023_42244_MOESM6_ESM.zip › Original data-final/Figure 2 a-l/(h)/1_m001.jpg]

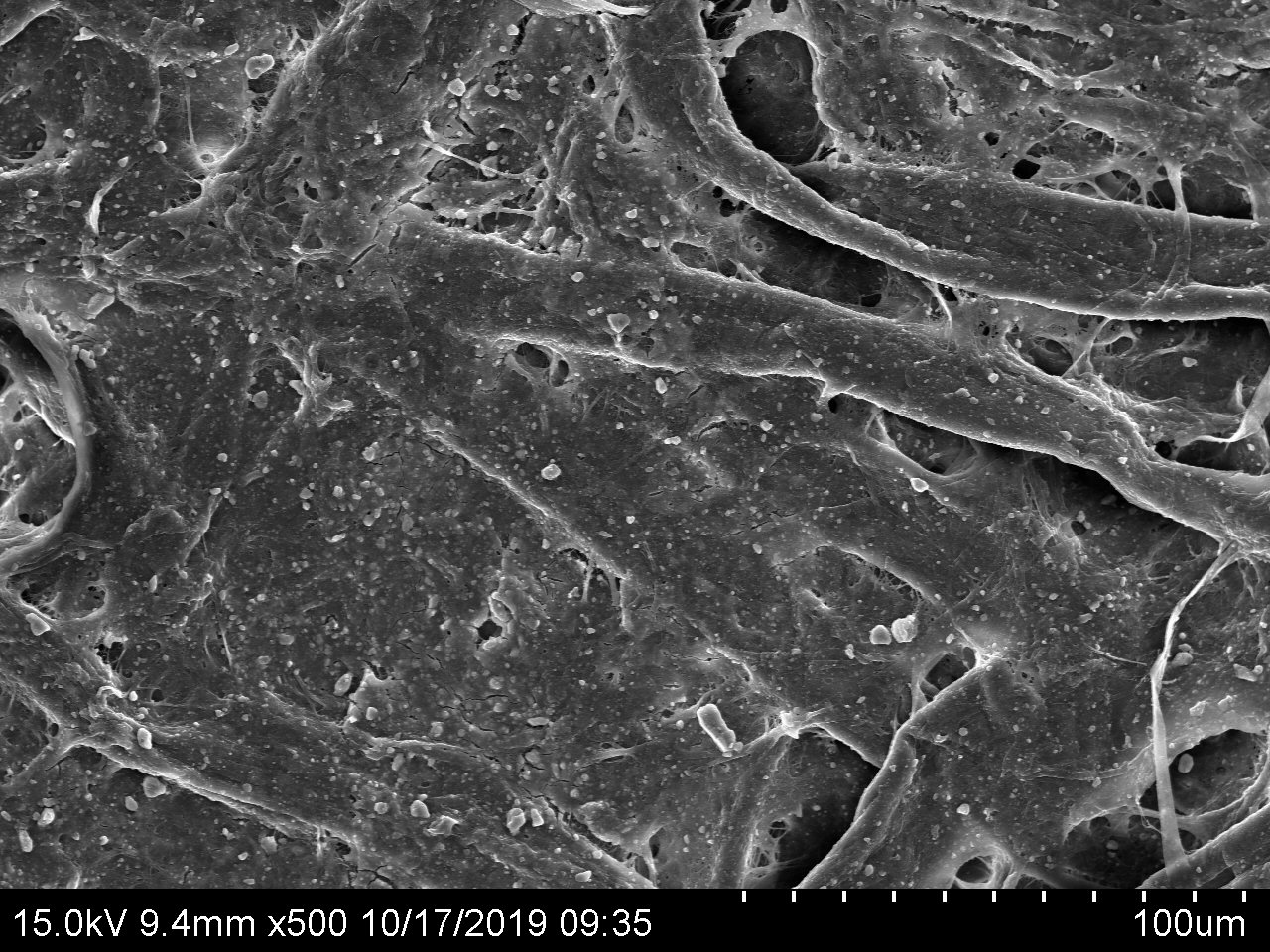

Supplement: Supplementary file 6 — Source Data [file 41467_2023_42244_MOESM6_ESM.zip › Original data-final/Figure 2 a-l/(i)/1_m001.jpg]

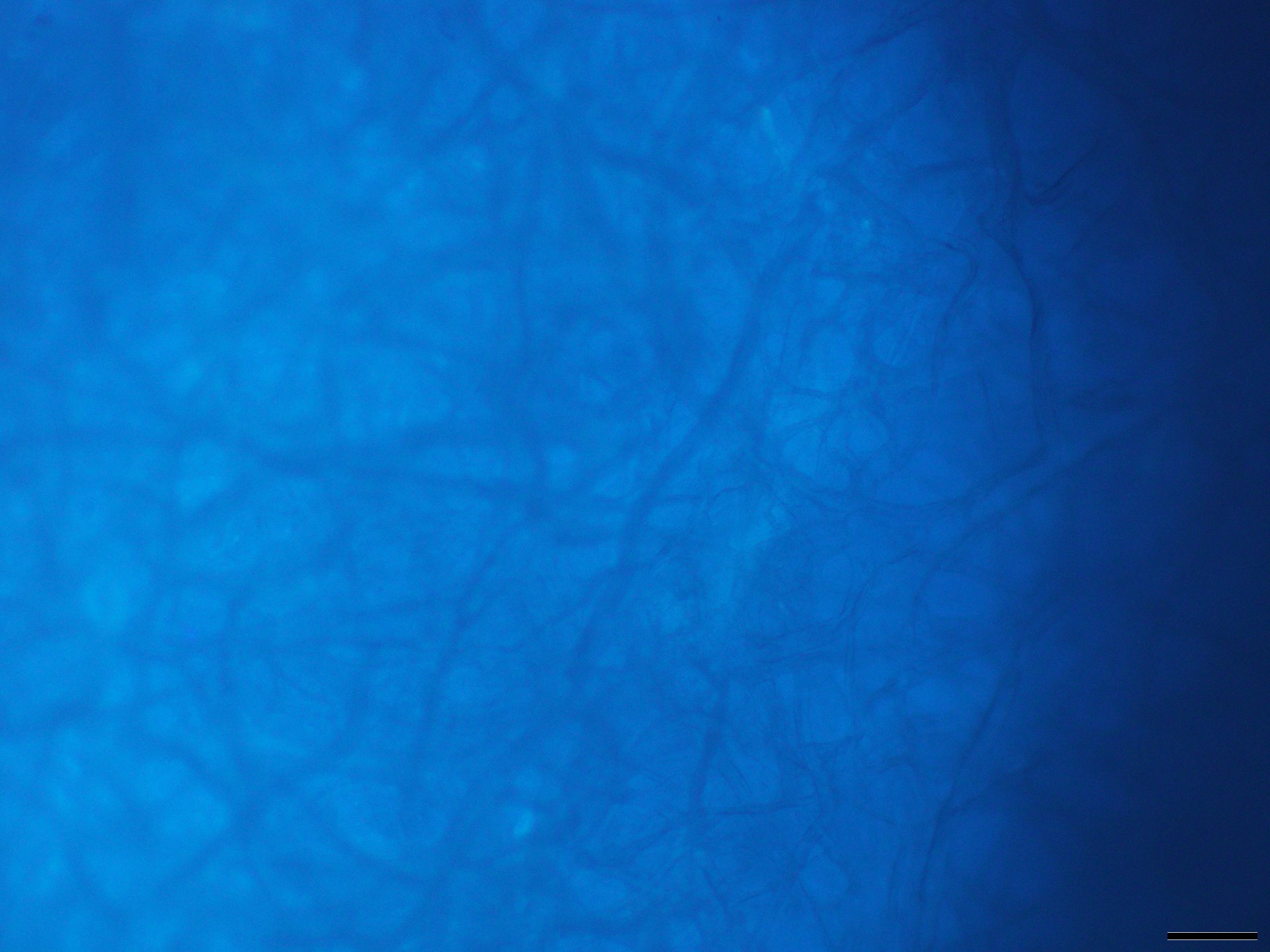

Supplement: Supplementary file 6 — Source Data [file 41467_2023_42244_MOESM6_ESM.zip › Original data-final/Figure 2 a-l/(j)/fig_5302.jpg]

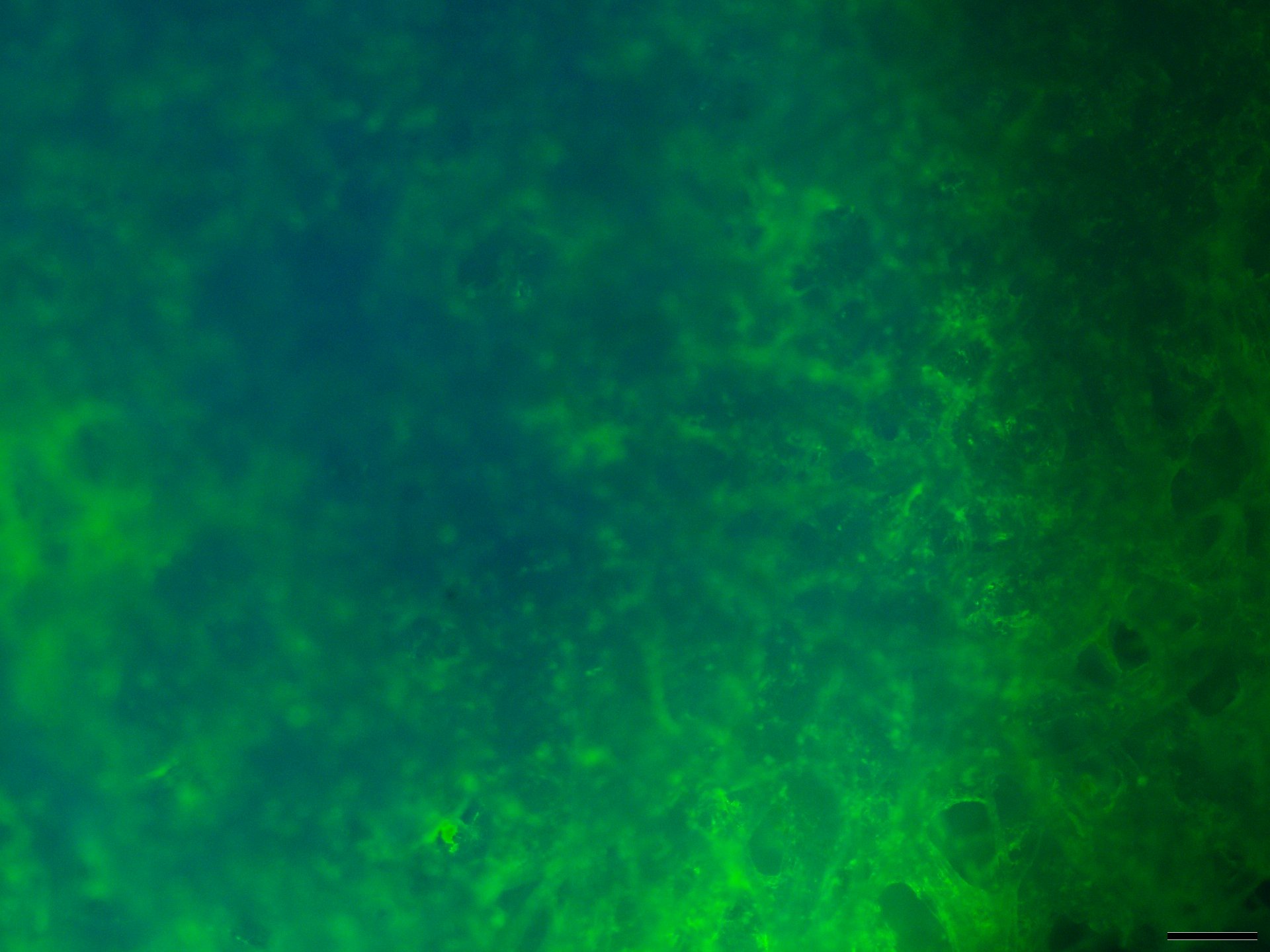

Supplement: Supplementary file 6 — Source Data [file 41467_2023_42244_MOESM6_ESM.zip › Original data-final/Figure 2 a-l/(k)/fig_5310.jpg]

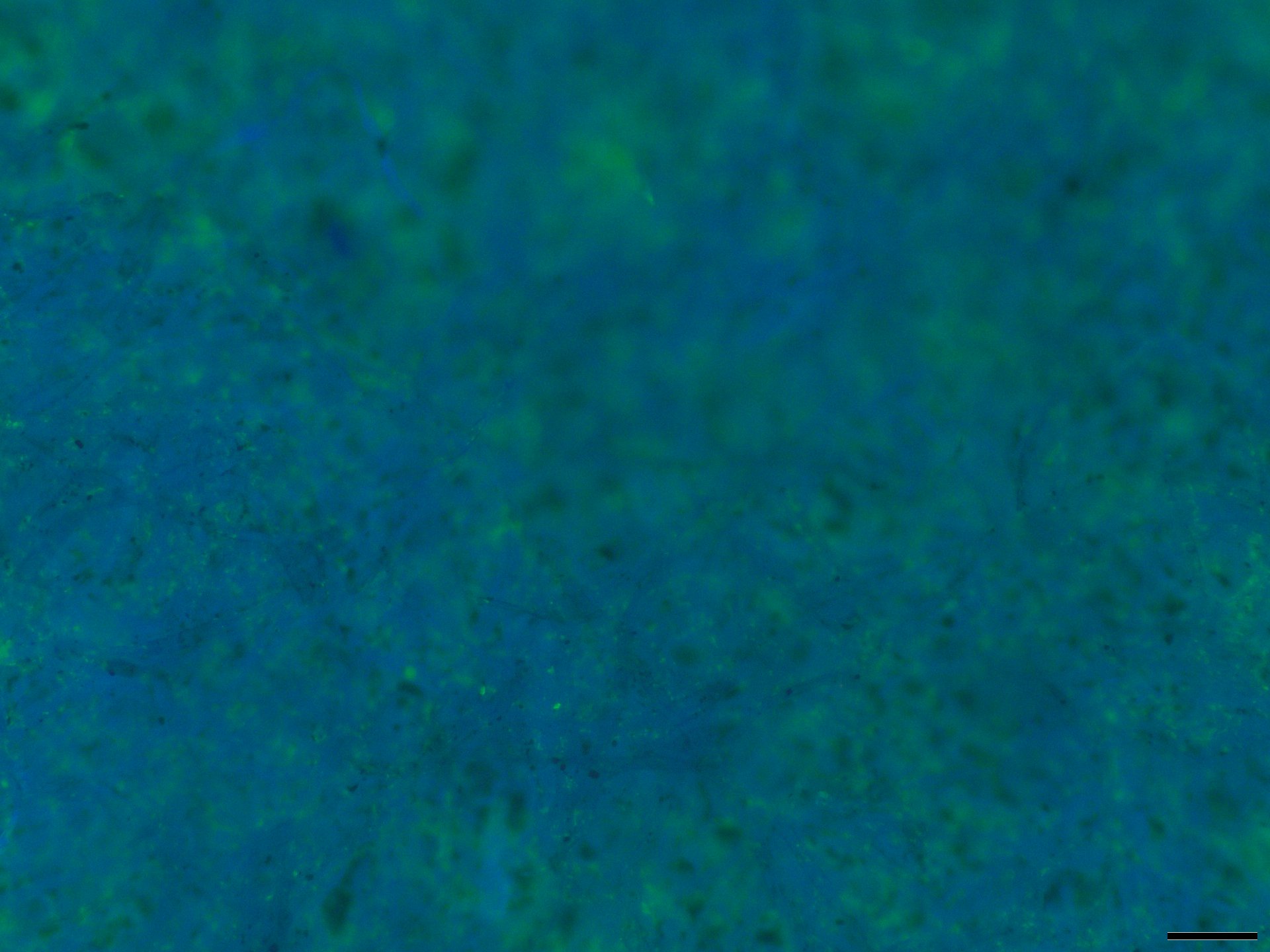

Supplement: Supplementary file 6 — Source Data [file 41467_2023_42244_MOESM6_ESM.zip › Original data-final/Figure 2 a-l/(l)/fig_5298.jpg]
